# Assessment of body composition in adults hospitalized with acute COVID-19: a scoping review

**DOI:** 10.3389/fnut.2023.1176441

**Published:** 2023-09-07

**Authors:** Isabel Pinto Amorim das Virgens, Iasmin Matias Sousa, Agnes Denise Lima Bezerra, Ana Paula Trussardi Fayh

**Affiliations:** ^1^Graduation Program in Health Sciences, Health Sciences Center, Federal University of Rio Grande do Norte, Natal, Rio Grande do Norte, Brazil; ^2^Center for Translational Medicine, Semmelweis University, Budapest, Hungary

**Keywords:** nutritional status, skeletal muscle mass, body fat, coronavirus disease 2019, hospitalized patient

## Abstract

**Introduction:**

Body composition (BC) assessment can supply accurate information for in-hospital nutritional evaluation. The aim of this study was to explore in the literature how the studies assessed BC, for what purpose, and investigate the role of BC findings in COVID-19 hospitalized patients’ outcomes.

**Methods:**

A scoping review was conducted according to the methodology available on the Joanna Briggs Institute website. We used the PCC acronym for the systematic search (population: adults with COVID-19, concept: assessment of BC, context: hospital setting) and performed it on PubMed, Scopus, and the Web of Science on 16 September 2022. Eligibility criteria consisted of the utilization of BC assessment tools in COVID-19 patients. Studies in which BC was solely measured with anthropometry (perimeters and skinfolds) were excluded. No language restriction was applied.

**Results:**

Fifty-five studies were eligible for the review. Out of the 55 studies, 36 used computed tomography (CT), 13 used bioelectrical impedance (BIA), and 6 used ultrasound (US). No studies with D3-creatinine, 24  h urine excretion, dual-energy X-ray absorptiometry, or magnetic resonance were retrieved. BC was mainly assessed to test associations with adverse outcomes such as disease severity and mortality.

**Discussion:**

Studies assessing BC in hospitalized patients with COVID-19 used mainly CT and BIA and associated the parameters with severity and mortality. There is little evidence of BC being assessed by other methods, as well as studies on BC changes during hospitalization.

## 1. Introduction

The coronavirus disease 2019 (COVID-19) has been, for over the past 3 years, the most serious public health emergency on several continents. On 11 March 2020, the World Health Organization (WHO) declared it a global pandemic (1). According to the WHO, by 23 April 2023, there were over 764 million confirmed cases and over 6.9 million deaths due to the disease worldwide (2). In this regard, some risk factors were found to be associated with COVID-19 severity and mortality. Obesity (body mass index >30 kg/m^2^) and/or high quantities of visceral adipose tissue (VAT) have been reported as predictors for hospitalization, severe state, and mortality in COVID-19 patients since they are linked with a high production of proinflammatory cytokines and an exacerbated inflammatory state (3–5).

Like obesity, reduced muscle mass (MM) or low skeletal muscle density (SMD) were found to be associated with worse prognosis in COVID-19 patients (6, 7). As COVID-19, like many other inflammatory diseases, has an impact on nutritional status due to the high consumption of protein and decreased protein synthesis (8, 9), changes in body composition (BC) might be exacerbated during the acute phase of the disease (10, 11). Therefore, BC assessment tools can be adopted to collect more accurate data on the presence of obesity as well as MM parameters.

Several BC assessment tools can be used to evaluate the adipose and muscle tissues in hospitalized patients, including at the bedside, and hence improve nutritional care and management. Image methods, such as computed tomography (CT), magnetic resonance imaging (MRI), dual-energy X-ray absorptiometry (DXA), ultrasound (US), and bioelectrical impedance (BIA), have been used for BC assessment in various clinical settings, due to their “opportunistic nature” during hospitalization (12). Abnormal BC as a predictor of negative outcomes is largely reported in some hospitalized populations, however, it has not yet been explored in COVID-19 patients (11). Thus, identifying how clinicians are currently assessing and monitoring BC in clinical settings is necessary for the implementation of adequate nutritional care.

Many observational studies investigated the predictive power of BC to assess the severity of COVID-19. The aim of this review was to identify the studies using parameters derived from BC assessment tools, report how the assessment was conducted, highlight the abnormalities in BC during hospitalization and summarize the main results. As the clinical question is broad and leads to other sub-questions, we chose to perform a scoping review. Thus, by identifying possible gaps in this topic, we can improve the research and the clinical practice regarding BC assessment in COVID-19 hospitalized patients as well as other hospitalized patients under acute inflammatory states.

## 2. Materials and methods

### 2.1. Study design

A scoping review was conducted, drawing inspiration from the Joanna Briggs Institute (JBI) (13). The PRISMA checklist for scoping reviews was filled out and is presented as Supplementary material (14). A review protocol was built for the scoping review, however, it was used only by our review team and not registered. This study analyzed qualitative and quantitative data presented in studies in which COVID-19 patients have undergone BC assessment.

### 2.2. Review question

From the available literature about BC assessment in COVID-19 hospitalized patients, what tools were utilized by the studies, and what are the gaps in the literature regarding BC assessment?

In this review, the acronym for population, concept, and context (PCC) for scoping reviews was as follows: Population (P) – adults older than 18 years hospitalized with COVID-19; Concept (C) – BC evaluated by non-anthropometric BC assessment tools; and Context (C) – hospital setting.

For this review, four research sub-questions were raised:

1. Regarding the tools, how did the studies with COVID-19 patients evaluate BC?
2. What were the objectives of the studies with COVID-19 patients submitted to BC assessment?
3. What were the main findings regarding BC parameters and COVID-19 prognosis?
4. What BC alterations occurred in patients with COVID-19 during hospitalization?

### 2.3. Eligibility criteria

All the studies evaluating hospitalized adults over 18 years of age with a diagnosis of COVID-19 and assessed by BC assessment tools were eligible for the scoping review. Our exclusion criteria consisted of non-targeted populations such as children, adolescents, pregnant women, and outpatients, studies that only assessed other body compartments such as epicardial fat thickness or the diaphragm muscle for cardiovascular and respiratory capacity assessment, and inappropriate study design, e.g., reviews, and case reports. No language restriction was applied, and only peer-reviewed, published data were eligible for inclusion. Although we focused on hospitalized patients, a few studies reported hospitalization as an outcome for outpatients (15, 16) and therefore they were also included.

### 2.4. Search strategy

A search strategy was constructed, and one reviewer (IPAV) systematically carried out the searches on electronic databases to find eligible articles published until 16 September 2022. The databases accessed were PubMed (accessed through the Medical Literature Analysis and Retrieval System Online MEDLINE), Web of Science, and Scopus. Subsequently, the titles and abstracts were exported to the citation manager EndNote software version 20.4.1 (Clarivate Analytics, Philadelphia, PA, United States) for manual duplicate removal. After the duplicate removal, the remaining references were shared among reviewers for the study selection. IPAV performed the title and abstract reading. The full-text assessment was performed by IPAV and IMS. In case of disagreements, ADLB decided whether the reference would be eligible for inclusion or not. Data extraction was performed by IPAV and checked by IMS. Again, in case of disagreements regarding the data extraction, a third reviewer was invited to resolve it (ADLB). We additionally carried out manual searches in reference lists of selected published studies to include eligible articles in case they were not available within the results yielded by the search strategy. No language nor time restriction was applied for our search. Additional contact with the authors was not necessary. The search key used on PubMed is presented in Box 1.

BOX 1 **Search strategy used on PubMed**

((Diagnostic Imaging[MeSH Terms]) OR (Imaging, Diagnostic[Title/Abstract])) OR (Medical Imaging[Title/Abstract])) OR (Imaging, Medical[Title/Abstract])) OR (Ultrasonography[MeSH Terms])) OR (Diagnostic Ultraso*[Title/Abstract])) OR (Ultraso* Imaging[Title/Abstract])) OR (Medical Sonography[Title/Abstract])) OR (Echography[Title/Abstract])) OR (Computer Echotomography[Title/Abstract])) OR (Ultrasonic Tomography[Title/Abstract])) OR (Diagnostic Techniques and Procedures[MeSH Terms])) OR (Diagnostic Testing[Title/Abstract])) OR (Tomography, X-Ray Computed[MeSH Terms])) OR (Tomography, X-Ray Computerized[Title/Abstract])) OR (X-Ray Computer Assisted Tomography[Title/Abstract])) OR (Computerized Tomography, X Ray[Title/Abstract])) OR (CT X Ray*[Title/Abstract])) OR (Tomography, X Ray Computed[Title/Abstract])) OR (Tomography, X Ray Computed[Title/Abstract])) OR (CAT Scan, X Ray[Title/Abstract])) OR (Tomography, Transmission Computed[Title/Abstract])) OR (CT Scan, X-Ray[Title/Abstract])) OR (Computed Tomography, X-Ray[Title/Abstract])) OR (computed tomography[Title/Abstract])) OR (magnetic resonance imaging[MeSH Terms])) OR (magnetic resonance imaging[Title/Abstract])) OR (NMR Imaging[Title/Abstract])) OR (Tomography, NMR[Title/Abstract])) OR (MR Tomography[Title/Abstract])) OR (Magnetic Resonance Image*[Title/Abstract])) OR (MRI Scan*[Title/Abstract])) OR (Absorptiometry, Photon[MeSH Terms])) OR (Photon Absorptiometry[Title/Abstract])) OR (X-Ray Densitometry[Title/Abstract])) OR (X-Ray Photodensitometry[Title/Abstract])) OR (Dual-Energy X-Ray Absorptiometry Scan[Title/Abstract])) OR (DXA Scan*[Title/Abstract])) OR (DEXA Scan*[Title/Abstract])) OR (Dual-Photon Absorptiometry[Title/Abstract])) OR (Dual-Energy Radiographic Absorptiometry[Title/Abstract])) OR (X Ray Absorptiometry[Title/Abstract])) OR (Dual Energy X Ray Absorptiometry[Title/Abstract])) OR (DPX Absorptiometry[Title/Abstract])) OR (Dual X-Ray Absorptiometry[Title/Abstract])) OR (Tomography, Emission Computed [Title/Abstract])) OR (Densitometry [Title/Abstract])) OR (imaging techniques[Title/Abstract])) OR (bioelectrical impedance analysis[Title/Abstract])) OR (BIA[Title/Abstract])) OR (bioimpedance analysis[Title/Abstract])) OR (bioelectrical impedance analysis[Title/Abstract])) OR (neutron-activation analysis[Title/Abstract]) OR (Electric Impedance[MeSH Terms])) OR (ultrasound[Title/Abstract]) OR (sonography[Title/Abstract]) OR (CT scan[Title/Abstract]) AND (“COVID-19”[Mesh] OR COVID 19 OR COVID-19 Virus Disease OR COVID 19 Virus Disease OR COVID-19 Virus Diseases OR Disease, COVID-19 Virus OR Virus Disease, COVID-19 OR COVID-19 Virus Infection OR COVID 19 Virus Infection OR COVID-19 Virus Infections OR Infection, COVID-19 Virus OR Virus Infection, COVID-19 OR 2019-nCoV Infection OR 2019 nCoV Infection OR 2019-nCoV Infections OR Infection, 2019-nCoV OR Coronavirus Disease-19 OR Coronavirus Disease 19 OR 2019 Novel Coronavirus Disease OR 2019 Novel Coronavirus Infection OR 2019-nCoV Disease OR 2019 nCoV Disease OR 2019-nCoV Diseases OR Disease, 2019-nCoV OR COVID19 OR Coronavirus Disease 2019 OR Disease 2019, Coronavirus OR SARS Coronavirus 2 Infection OR SARS-CoV-2 Infection OR Infection, SARS-CoV-2 OR SARS CoV 2 Infection OR SARS-CoV-2 Infections OR COVID-19 Pandemic OR COVID 19 Pandemic OR COVID-19 Pandemics OR Pandemic, COVID-19 OR “SARS-CoV-2”[Mesh] OR Coronavirus Disease 2019 Virus OR 2019 Novel Coronavirus OR 2019 Novel Coronaviruses OR Coronavirus, 2019 Novel OR Novel Coronavirus, 2019 OR Wuhan Seafood Market Pneumonia Virus OR SARS-CoV-2 Virus OR SARS CoV 2 Virus OR SARS-CoV-2 Viruses OR Virus, SARS-CoV-2 OR 2019-nCoV OR COVID-19 Virus OR COVID 19 Virus OR COVID-19 Viruses OR Virus, COVID-19 OR Wuhan Coronavirus OR Coronavirus, Wuhan OR SARS Coronavirus 2 OR Coronavirus 2, SARS OR Severe Acute Respiratory Syndrome Coronavirus) AND ((sarcopenia[Title/Abstract])) OR (sarcopenic obesity[Title/Abstract])) OR (Skeletal Muscle*[Title/Abstract])) OR (Voluntary Muscle*[Title/Abstract])) OR (Soleus Muscle[Title/Abstract])) OR (Plantaris Muscle[Title/Abstract])) OR (Anterior Tibial Muscle[Title/Abstract])) OR (Gastrocnemius Muscle[Title/Abstract])) OR (Muscle*[Title/Abstract])) OR (Muscle Tissue*[Title/Abstract])) OR (Skeletal muscle cutoff values[Title/Abstract])) OR (appendicular lean soft tissue[Title/Abstract])) OR (skeletal muscle mass[Title/Abstract])) OR (skeletal muscle area[Title/Abstract])) OR (skeletal muscle mass index[Title/Abstract])) OR (appendicular skeletal muscle mass index[Title/Abstract])) OR (fat-free mass index[Title/Abstract])) OR (muscle mass[Title/Abstract])) OR (Quadriceps Muscle*[Title/Abstract])) OR (Quadriceps Femoris[Title/Abstract])) OR (Vastus Medialis[Title/Abstract])) OR (Vastus Intermedius[Title/Abstract])) OR (Rectus Femoris[Title/Abstract])) OR (Vastus Lateralis[Title/Abstract])) OR (appendicular lean mass[Title/Abstract])) OR (appendicular skeletal muscle mass[Title/Abstract])) OR (Appendicular lean tissue mass[Title/Abstract])) OR (body surface area[Title/Abstract])) OR (fat-free mass[Title/Abstract])) OR (third lumbar vertebra[Title/Abstract])) OR (total abdominal muscle area[Title/Abstract])) OR (thigh muscle area[Title/Abstract]))) OR (psoas muscle index[Title/Abstract])) OR (psoas muscle area[Title/Abstract])) OR (body skeletal muscle mass[Title/Abstract])) OR (muscle indices[Title/Abstract])) OR (lean mass measures[Title/Abstract])) OR (lean mass[Title/Abstract])) OR (muscle tissue[Title/Abstract])) OR (Muscle wasting[Title/Abstract])) OR (muscle size[Title/Abstract]) OR (Body Fat Distribution[Title/Abstract])) OR (Adiposity [Title/Abstract]) OR (Body Constitution [Title/Abstract])) OR (Body Composition[Title/Abstract]) OR (Fatty Tissue[Title/Abstract])) OR (Adipose Tissue[Title/Abstract]) OR (Abdominal Fat[Title/Abstract])) OR (Abdominal Adipose Tissue[Title/Abstract]) OR (Subcutaneous Fat [Title/Abstract])) OR (Subcutaneous Adipose Tissue[Title/Abstract]) OR (phase angle[Title/Abstract]) OR (intramuscular adipose[Title/Abstract]) OR (muscle quantity[Title/Abstract]) OR (Subcutaneous fat area[Title/Abstract]) OR (visceral Adipose Tissue[Title/Abstract]) OR (visceral fat[Title/Abstract]).

The search strategy performed on Scopus and Web of Science is available in Supplementary material 1.

### 2.5. Data collection and charting

To address the research questions, the following data were extracted from each included study: (i) first author, year of publication, and journal; (ii) country and language; (iii) study design; (iv) population characteristics (sample size; sex; age and health status); (v) aim of the research paper; (vi) sample size estimation; (vii) main results of the study; (viii) type of BC assessment tool; (ix) moment of the assessment; (x) frequency of the assessment; (xi) report of the tool performer and report of the assessor of the BC tool; (xii) body markers/compartments measured; (xiii) report of the protocol; (xiv) exclusion criteria; (xv) criteria for the classification of the markers of BC; and (xvi) results of the BC assessment when available. After curating the information, the data were extracted to an Excel sheet and later exported and standardized into three tables (Tables 1–3). The first table summarizes the main characteristics of the studies, the second gives further information on the BC assessment, and the third provides the quantitative findings derived from the BC assessment.

**Table 1:** Characteristics of the studies evaluating body composition in COVID-19 patients.

Reference, journal and year of publication	Country and written language	Study design	Study sample	Assessment tool	Aim of the study	Report of sample size estimation	Main results
Del Giorno et al. (17)International Journal of General Medicine2020	Switzerland	Single-center retrospective cohort study	90 hospitalized patients with a mean age of 64.5 ± 13.7 years (male = 67.8%).	BIA	To investigate the associations between nutritional risk (by the NRS 2002), BIA data, and clinical outcomes.	No	BIA did not add further predictive value for death, admission at ICU, prolonged LOS, or loss of appetite.
	English						
Cornejo-Pareja et al. (18)Clinical Nutrition2021	Spain	Single-center prospective cohort study	127 adult hospitalized patients with a median age of 69.0 (IQR: 59.0–80.0) years (male = 59.1%).	BIA	To determine the predictive role of PhA on 90 days survival of adults.	Yes	Low PhA (<3.95°) was an independent predictor of mortality.
	English						
Da Porto et al. (19)Nutrients 2021	Italy	Single-center prospective observational study	150 hospitalized patients with a median age of 69.0 (IQR: 58.0–78.0) years (male = 68.7%).	BIA	To assess the prevalence of malnutrition utilizing BIVA and evaluate its relationships with severity and the outcomes of the disease.	No	Malnutrition according to BIVA was independently associated with a greater need of invasive MV and increased mortality in the short-term.
	English						
Kellnar et al. (20)Clinical Nutrition ESPEN 2021	Germany	Single-center prospective pilot study	12 ward patients with a median age of 70.6 (IQR: 49.5–72.9) years (male = 66.7%).	BIA	To investigate if COVID-19 infection was significantly associated with changes in BC during the hospital stay.	No	The pilot study found a significant decrease in body cell mass and PhA during the active infection of COVID-19 and a slow rehabilitation to the baseline characteristics toward discharge.
	English						
Moonen et al. (21)Clinical Nutrition2021	The Netherlands	Single-center cross-sectional cohort study	54 hospitalized patients with a mean age of 67.0 (CI: 64.0–71.0) years (male = 63.0%).	BIA	To assess the BC of patients admitted to the ward or the ICU and identify associations with disease severity.	No	Only a low PhA was shown to increase the odds of disease severity (ICU admission, morbidity, and mortality) in patients with COVID-19. BC measurements were not found to be risk factors for disease severity.
	English						
Moonen et al. (22)Clinical Nutrition ESPEN 2021	The Netherlands	Single-center prospective observational study	150 hospitalized patients with a median age of 68.0 (CI: 66.0–70.0) years (male = 67.0%).	BIA	To investigate the associations between baseline BC parameters and adverse outcomes after 90 days.	No	The increased odds of morbidity, ICU-admission, and mortality were significantly associated with a lower PhA.
	English						
Cornejo-Pareja et al. (23)Nutrients 2022	Spain	Single-center, prospective cohort study	127 hospitalized patients with a median age of 69.0 (IQR: 59.0–80.0) years (male = 59.1%).	BIA	To determine the predictive value of hydration status on 90-day survival.	Yes	Overhydration characterized by ECW/TBW >0.58 and hydration >76.15% were predictors of mortality.
	English						
Hegde et al. (24)Asia Pacific Journal of Clinical Nutrition2022	India	Prospective cohort study	172 hospitalized patients with a mean age of 51.0 ± 13.0 (male = 65.0%).	BIA	To evaluate the associations between percentage of FM and anthropometric measures with severity at admission and disease progression during hospitalization.	No	Body FM (%) was a good risk indicator to predict LOS and disease severity at admission.
	English						
Moonen et al. (9)Clinical Nutrition ESPEN 2022	The Netherlands	Post-hoc sub-study from a single-center, prospective cohort study	150 hospitalized patients with a median age of 68.0 (CI: 66.0–70.0) years (male = 67.0%)^*^	BIA	To explore which method agrees better with LM as measured by BIA.	No	Authors could not identify a mathematical method for the estimation of LM that agreed with LM measurement as derived from BIA.
	English		^*^The same cohort as the BIAC-19 prospective study.				
Osuna-Padilla et al. (25)Journal of Parenteral and Enteral Nutrition2022	Mexico	Single-center prospective cohort study	67 critically ill patients with a mean age of 55.3 ± 13.6 years (male = 76.0%).	BIA	To describe the associations between PhA by BIA with days on MV, LOS, and 60-day mortality.	Yes	Low PhA was associated with 60-day mortality.
	English						
Reyes-Torres et al. (26)Nutrition in Clinical Practice 2022	Mexico	Multicenter (two centers) prospective cohort study.	112 post-ICU patients with a mean age of 54.0 ± 12.0 years (male = 82.0%).	BIA	To assess the BC and prevalence of post-extubation dysphagia in patients discharged from an ICU.	No	Overhydration and low PhA were associated with the presence of dysphagia. Lower PhA was an independent factor for impaired swallowing recovery at ICU discharge.
	English						
Ryrsø et al. (27)International Journal of Obesity 2022	Denmark	Single-center, prospective cohort study	40 hospitalized patients with a median age of 72.0 (IQR: 59.0–77.0) years (male = 60.0%).	BIA	To explore differences in BC, metabolic profile, inflammation, and physical capacity between patients hospitalized with community acquired pneumonia due to different pathogens.	No	FFM, FM, and BMI were similar between groups.
	English						
Stevanovic et al. (33)Frontiers in Nutrition2022	Serbia	Prospective cohort study	216 hospitalized patients with a median age of 67.0 (IQR: 17.75) years (male = 63.0%).	BIA	To investigate the impact of visceral and body fat on COVID-19 outcomes.	No	Obesity defined by BIA parameters was associated with ICU admission and mortality.
	English						
Andrade-Júnior et al. (28)Frontiers in Physiology 2021	Brazil	Single-center prospective cohort study	32 critically ill patients with a mean age of 64.1 ± 12.6 years (male = 93.8%).	US	To characterize and evaluate functional performance and MM in intensive care patients.	No	Patients in a severe state had a reduction both in the cross-sectional rectus femoris muscle area and in the thickness of the anterior compartment of the quadriceps.
	English						
Bologna and Pone (29)Healthcare (Basel)2022	Italy	Parallel randomized study	80 patients of which 40 were in the control group and 40 were in the supplementation group. No data on age nor sex was reported.	US	To evaluate the efficacy of a 3 g arginine supplementation/day blended with other nutrients and its association with the treatment and prevention of sarcopenia.	No	The intervention group had improved muscular and respiratory performance compared with the control group.
	English						
Formenti et al. (30)Journal of Critical Care 2021	Italy	Single-center prospective observational study	32 critically ill patients who undergone intubation with a mean age of 63.9 ± 7.4 years (male = 78.0%).	US	To investigate the characteristics of the respiratory and peripheral muscles of patients affected by the disease in MV evaluated by US.	Yes	Greater values of echogenicity of the rectus femoris, diaphragm, and right intercostal sites were associated with mortality.
	English						
Gil et al. (31)Journal of Cachexia, Sarcopenia, and Muscle 2021	Brazil	Single-center prospective observational study	186 hospitalized patients with a mean age of 59.0 ± 15.0 years (male = 50.0%).	US	To investigate if MM or muscle strength predicts LOS in patients with moderate to severe disease.	Yes	MM along with muscle strength were predictors of LOS in patients with moderate to severe COVID-19.
	English						
Umbrello et al. (6)Nutrition 2021	Italy	Single-center prospective observational study	28 critically ill patients in invasive MV with a mean age of 65.0 ± 10.0 years (male = 80.0%).	US	To compare the size and quality of the diaphragm and rectus femoris muscles between the critically ill, COVID-19 survivors, and non-survivors during hospitalization.	Yes	Early changes in muscle parameters seem to be related to the outcome of critically ill COVID-19 patients.
	English						
Kremer et al. (32)Journal of Cachexia, Sarcopenia, and Muscle 2022	Germany	Single-center prospective observational study	113 hospitalized patients with a median age of 69.0 (IQR: 57.0–79.0) years (male = 69.1%).	US	To explore muscle indices evaluated by US as predictors of COVID-19 outcome as well as to test the feasibility of the tool in an isolated context.	Yes	There was significantly greater mortality in the group with PMAI and PMTI below the gender-specific medians in the 30-day follow-up.
	English						
Battisti et al. (34)Diabetes Care 2020	Italy	Single-center prospective cohort study	144 hospitalized patients with a mean age of 60.3 ± 17.0 (male = 60.4%).	CT	To assess the relationship between the severity of the disease and abdominal fat distribution.	No	Increased risk for ICU admission was associated with abdominal adipose tissue distribution (higher VAT and lower SAT).
	English						
Favre et al. (35)Metabolism Clinical and Experimental2020	France	Prospective cohort study	165 hospitalized patients with a mean age of 64.0 ± 17.0 years (66.1% male).	CT	To show that VAT better predicts the severity of COVID-19 outcome compared to either SAT or BMI.	No	VAT was significantly associated with the severity of the disease. A VAT area ≥ 128.5 was found to be the best predictive value for severe COVID-19.
	English						
Gualtieri et al. (36)International Journal of Molecular Sciences2020	Italy	Single-center retrospective cohort study	30 hospitalized patients with a mean age of 55.4 ± 12.5 years (63.3% male).	CT	To evaluate the contrast in BC overall, lean, and obese groups during ICU hospitalization.	Yes	Loss of LM index and FM was observed in the first 20 days of hospitalization. An increase in liver attenuation was observed in patients with obesity.
	English						
Kottlors et al. (37)European Journal of Radiology 2020	Germany	Multicenter (two centers) retrospective cohort study	58 hospitalized patients with a mean age of 59.3 ± 16.2 years (male = 63.8%).	CT	To investigate whether the FMR determined by low dose CT can predict severe progression of the disease.	No	FMR was significantly higher in the group of patients requiring ICU treatment.
	English						
Petersen et al. (38)Metabolism Clinical and Experimental2020	Germany	Single-center cross-sectional study	30 hospitalized patients with a mean age of 65.6 ± 13.1 years (male = 60.0%).	CT	To investigate the association between the severity of the disease and adipose tissue distribution.	No	Greater quantities of VAT were significantly associated with the increased probability of severe illness.
	English						
Watanabe et al. (39)Metabolism Clinical and Experimental2020	Italy	Single-center retrospective cohort study	150 hospitalized patients with a mean age of 64.0 ± 16.0 years (male = 64.7%).	CT	To explore the impact of abdominal fat as a marker of BC on disease severity.	Yes	Accumulation of VAT was higher in ICU patients when compared with homecare and sub-intensive care patients.
	English						
Yang et al. (40)Obesity 2020	China	Single-center retrospective cohort study	143 hospitalized patients with a median age of 66.0 (IQR: 56.0–73.5) years (male = 49.0%).	CT	To assess the association between the distribution of adipose tissues and the disease severity during hospitalization.	No	VAT and high IMAT were independent risk factors for critical illness.
	English						
Besutti et al. (41)PLOS One 2021	Italy	Retrospective cohort study. Number of centers not specified.	318 hospitalized patients with a median age of 65.7 (IQR: 52.8–75.7) years (male = 53.3%).	CT	To investigate the association between BC parameters derived from CT and clinical outcomes (hospitalization, MV, and mortality).	No	Higher SMD was shown to be a protective factor for hospitalization, MV, and death. Contrarily, increased VAT, IMAT, and TAT were risk factors for these outcomes.
	English						
Bunnell et al. (42)International Journal of Obesity 2021	United States of America	Single-center retrospective cohort study	124 hospitalized patients with a median age of 68.0 (IQR: 56.0–77.0) years (male = 52.4%).	CT	To evaluate BC by CT as a predictor of outcome in hospitalized patients.	No	IMAT and VAT/SAT ratio were associated with a higher risk of death or ICU admission.
	English						
Chandarana et al. (15)European Journal of Radiology 2021	United States of America	Multicenter (two centers) retrospective cohort study	177 hospitalized patients with a mean age of 59.0 ± 16.0 years (male = 55.0%).	CT	To assess the prognostic value of BC parameters to predict risk of hospitalization.	No	A significant difference was found in the MAT and IMAT/MM biomarkers between hospitalized and non-hospitalized patients.
	English						
Chandarana et al. (16)Abdominal Radiology2021	United States of America	Retrospective cohort study	51 hospitalized (n = 41) with a mean age of 60.8 ± 15.8 years and outpatients (n = 10) with a mean age of 54.7 ± 11.6 years (male = 74.5%).	CT	To assess SAT, VAT, and TAT estimations at the abdominopelvic levels derived from CT.	No	Higher values of VAT area were observed in hospitalized COVID-19 patients when compared with the group of outpatients.
	English						
Damanti et al. (43)Clinical Nutrition2021	Italy	Single-center retrospective cohort study.	81 critically ill patients with a mean age of 59.3 ± 11.91 years (male = 87.7%).	CT	To evaluate the associations between MM and quality in predicting complications, LOS, length of ICU stay, and mortality in patients admitted to ICU.	Yes	ICU length of stay was influenced by SMI, as well as complications in the ICU. Muscle area was a predictor of complications for patients in the ICU.
	English						
Feng et al. (44)Journals of Gerontology Series A: Biological Sciences and Medical Sciences 2021	China	Multicenter (five centers) retrospective cohort study	116 patients with severe COVID-19 with a median age of 57.0 (IQR: 29.0–84.0) years (male = 54.3%).	CT	To determine the associations between clinical outcomes and skeletal muscle depletion.	No	Higher PMD was associated with a decreased risk of disease deterioration and inferior likelihood of longer viral shedding in female patients.
	English						
Giraudo et al. (7)PLOS One 2021	Italy	Secondary analysis study	150 hospitalized patients with a mean age of 61.3 ± 15.0 years (male = 69.3%).	CT	To assess if reduced MM is a predictor of ICU admission in hospitalized patients.	No	Patients that were admitted to ICU had significantly lower MM values.
	English						
Goehler et al. (45)Open Forum Infectious Diseases2021	United States of America	Single-center retrospective cohort study	378 hospitalized patients with a mean age of 63.3 ± 17.8 years (male = 61.7%).	CT	To test whether VAT is associated with severe outcomes.	No	Increased VAT was associated with a higher risk of severe disease or mortality. Individuals with higher VAT were more likely (twice the risk) of being intubated or dying when compared with the patients with normal VAT.
	English						
Hoyois et al. (46)JPEN Journal of Parenteral and Enteral Nutrition 2021	Belgium	Single-center prospective cohort study	15 ICU patients with a median age of 60.0 (IQR: 33.0–75.0) years (male = 67.0%).	CT	To assess the nutritional status and outcomes in patients following ICU discharge.	No	Critically ill patients had low MM and malnutrition at discharge.
	English						
McGovern et al. (47)The Journal of Nutrition 2021	United Kingdom	Single-center cross-sectional study	63 hospitalized patients (60.0% ≥ 70 years; male = 47.3%).	CT	To assess the relationship between BC measurements derived from CT measurements, systemic inflammation, and clinical outcomes.	No	Sarcopenia defined by SMI thresholds in the presence of obesity (defined by BMI) was associated with greater 30-d mortality.
	English						
Moctezuma-Velázquez et al. (48)American Journal of Physical Medicine and Rehabilitation 2021	Mexico	Single-center retrospective cohort study	519 hospitalized patients with a median age of 51.0 (IQR: 42.0–61.0) years (male = 64.0%).	CT	To verify the associations between in-hospital mortality, ICU admission, and use of invasive MV and low SMI.	No	ICU admission, need for invasive MV, and mortality were not associated with low SMI.
	English						
Nobel et al. (49)Digestive Diseases and Sciences 2021	United States of America	Single-center retrospective cohort study	190 hospitalized patients with a median age of 66.0 (IQR: 51.0–74.0) years (male = 55.6%) divided into two groups (with or without gastrointestinal symptoms).	CT	To determine if unfavorable BC biomarkers are associated with adverse outcomes among patients with gastrointestinal symptoms.	No	Patients without gastrointestinal symptoms presented expected associations between BC and worse outcomes: higher mortality in those with low SMI, and high IMAT, as well as a higher VAT/SAT ratio. The group who had gastrointestinal symptoms did not.
	English						
Ogata et al. (50)BMC Infectious Diseases 2021	Japan	Single-center retrospective cohort study	53 hospitalized patients with a mean age of 60.0 years ±20.0 years (male = 62.3%).	CT	To investigate if intra-abdominal fat is useful to predict disease prognosis.	No	An increased VAT/TAT ratio was an independent risk factor for disease severity in hospitalized patients.
	English						
Pediconi et al. (51)Obesity Research and Clinical Practice2021	Italy	Multicenter (two centers) retrospective cohort study	62 hospitalized patients with a mean age of 70.0 ± 14.0 years (male = 64.5%).	CT	To assess the relationship between SAT and VAT with lung disease severity as well as to test their potential to predict ICU admission.	No	VAT was found to be the best predictor for ICU admission. VAT and SAT were also significantly correlated to lung disease severity.
	English						
Polat et al. (52)Turkish Journal of Geriatrics 2021	Turkey	Single-center prospective cohort study	130 hospitalized patients with a median age of 74.0 (IQR: 68.0–79.0) years (male = 100%).	CT	To assess the associations between sarcopenia assessed by the psoas muscle and disease prognosis in male adults.	No	Psoas measurements added predictive value for the prognosis of COVID-19.
	English						
Poros et al. (53)Obesity Medicine 2021	Germany	Single-center retrospective cohort study	74 hospitalized patients with a median age of 66.0 (IQR: 57.0–72.8) years (male = 81%); 67 patients with CT scans.	CT	To determine if the anthropometric markers of abdominal VAT and thoracic skeletal muscle correlate with worse outcomes.	No	Worse outcomes in the patients with critical illness were associated with reduced thoracic MM and higher values of abdominal VAT.
	English						
Rossi et al. (54)Frontiers in Physiology 2021	Italy	Single-center cross-sectional study	153 ICU patients with a mean age of 64.2 ± 9.98 (male = 79.1%).	CT	To determine if different IMAT are associated with mortality and muscle damage in patients affected by the disease admitted to the ICU.	No	ICU patients with higher values of IMAT and low SMD were at higher risk of ICU mortality and muscle injury.
	English						
Scheffler et al. (55)Clinical Medicine2021	Switzerland	Single-center retrospective cohort study	64 octogenarian patients with a mean age of 86.4 ± 6.0 years (male = 46.9%).	CT	To investigate the association between VAT and SAT and in-hospital mortality.	No	Higher values of SAT had a positive effect against mortality in this sample, even when adjusted for sex, BMI, and age. On the contrary, higher VAT, TAT, and abdominal circumference were associated with worse COVID-19 pneumonia.
	English						
Schiaffino et al. (56)Radiology 2021	Italy	Multicenter (four centers) retrospective cohort study	552 hospitalized patients with a median age of 65.0 years (IQR: 54.0–75.0; male = 65.9%).	CT	To investigate whether the muscle parameters status derived from CT predicted adverse clinical outcomes.	No	In-hospital mortality and admission to the ICU were independently associated with lower MM.
	English						
Viddeleer et al. (57)Journal of Cachexia, Sarcopenia, and Muscle 2021	The Netherlands	Prospective cohort study	215 hospitalized patients with a mean age of 61.1 ± 14.3 years (male = 60.0%).	CT	To examine the association between BC measures and survival.	No	Higher IMAT was significantly associated with mortality in COVID-19 patients.
	English						
Antonarelli et al. (58)Tomography 2022	Italy	Single-center retrospective cohort study	112 hospitalized patients with a mean age of 60.5 ± 11.4 years were included (male = 73.2%).	CT	To evaluate the association between the chest CT-derived muscle analysis of sarcopenia and clinical-radiological outcomes.	No	Decreased pectoralis muscle area could add further predictive value for ICU stay and successful extubation. However, both pectoralis muscle and density could not predict risk of mortality or pneumonia severity.
	English						
Attaway et al. (59)Journal of Cachexia, Sarcopenia, and Muscle 2022	United States of America	Multicenter retrospective cohort study (Cleveland Clinic main campus and regional facilities)	95 hospitalized patients with a mean age of 63.3 ± 14.3 years (male = 52.6%).	CT	To determine the rate of MM loss and its association with adverse clinical outcomes.	No	Acute sarcopenia characterized by reductions of both pectoralis and erector spinae muscles was associated with adverse clinical outcomes.
	English						
Beltrão et al. (60)Endocrine Connections 2022	Brazil	Single-center prospective cohort study	200 moderately to severely ill patients with a median age of 62.0 (IQR: 50.0–74.0) years (male = 52.0%).	CT	To analyze the associations between clinical outcomes and BC findings.	Yes	Low MM area, high VAT, and VAT/MA ratios were independent predictors for mortality.
	English						
Bodolea et al. (61)Nutrients 2022	Romania	Single-center retrospective cohort study	90 patients with severe disease and acute respiratory distress syndrome with a median age of 67.0 (IQR: 36.0–89.0) years (male = 58.9%).	CT	To evaluate the function of four nutritional risk assessment instruments^*^ together with CT-derived adipose tissue and MM in predicting in-hospital mortality.	No	No statistical difference was found between survivors and deceased patients regarding measurements of BC.
	English						
Do Amaral e Castro et al. (62)Journal Einstein 2022	Brazil	Single-center retrospective cohort study	123 hospitalized patients with a mean age of 57.4 ± 16.5 years (male = 64.2%).	CT	To evaluate BC and clinical data derived from CT and verify its association with disease severity.	No	No statistical difference was found between the worse outcome and better outcome groups regarding the measurements of BC.
	English						
Faiella et al. (63)Journal of Clinical Medicine Research 2022	Italy	Single-center retrospective cohort study	132 hospitalized patients divided in two groups, bleeding group (*n* = 70) and control group (n = 62), with mean ages of 70.9 ± 11.6 (male = 50.0%) and 65.0 ± 11.2 (male = 46.0%), respectively.	CT	To analyze the relationship between quantities of adipose tissue derived from CT, BC measurements, and patient characteristics, and incidence of soft tissue bleeding requiring medical intervention.	No	Soft tissue bleeding was more severe and frequent in patients with low quantities of VAT.
	English						
McGovern et al. (64)Journal of Translational Medicine 2022	United Kingdom	Single-center cross-sectional study	63 hospitalized patients (60.0% ≥ 70 years; male = 47.3%).	CT	To assess the relationship between BC measurements derived from CT measurements, systemic inflammation, and clinical outcomes.	No	Sarcopenia defined by SMI thresholds in the presence of obesity (defined by BMI) was associated with greater 30-d mortality.
	English						
Menozzi et al. (65)Clinical Nutrition ESPEN 2022	Italy	Single-center retrospective cohort study	272 hospitalized patients with a median age of 71.0 (IQR: 61.0–78.0) years (male = 62.9%).	CT	To assess the prognostic role of sarcopenia in COVID-19 cohorts from the first wave and second wave.	No	A prognostic impact of sarcopenia in COVID-19 was found in the first wave cohort.
	English						
Molwitz et al. (66)Scientific reports 2022	Germany	Single-center retrospective cohort study	46 hospitalized patients with a mean age of 64.4 years ±11.4 (male = 58.7%).	CT	To investigate the relationship between thoracic (T12) and abdominal CT (L3) BC parameters to investigate sarcopenia/obesity.	No	T12 derived scans can be utilized to predict muscle parameters and abdominal fat.
	English						

BC, body composition; BIA, bioelectrical impedance; US, ultrasound; BIVA, bioelectrical impedance vector analysis; BMI, body mass index; CT, computed tomography; ICU, intensive care unit; MV, mechanical ventilation; ECW, extracellular water; ECW/TBW, extracellular water/total body water; FFM, fat free mass; FM, fat mass; FMR, muscle to fat ratio; IMAT, intermuscular adipose tissue; LM, lean mass; LOS, length of hospital stay; MAT, muscular adipose tissue; MAT/MM, muscular adipose tissue/muscle mass; MM, muscle mass; NRS 2002, Nutrition Risk Screening 2002; PhA, phase angle; PMAI, psoas muscle area index; PMTI, psoas muscle thickness index; SAT, subcutaneous adipose tissue; SMI, skeletal mass index; TAT, total adipose tissue; TBW, total body water; VAT, visceral adipose tissue; VAT/SAT, visceral adipose tissue/subcutaneous adipose tissue; ^*^PNI, Prognostic Nutritional Index; CONUT, Controlling Nutritional Status Score; NUTRIC, Nutrition Risk in Critically Ill; mNUTRIC, modified NUTRIC. Quantitative values are expressed in median and interval quartile range (IQR); mean and standard deviation (±), relative frequency (%), and confidence interval (CI).

**Table 2:** General characteristics about the body composition tool, measurements, and parameters utilized.

References	Body composition assessment tool used	Moment	Frequency	Evaluator	Body markers measured	Protocol	Exclusion criteria	How was it classified?
Del Giorno et al. (17)	BIA 101 (Akern Bioresearch, Florence, Italy).	Within 24 h after admission.	Once	Experienced dieticians	FFM, FM, BCM, TBW, and PhA.	Protocol reported. Values extracted from software recommended by the manufacturer.	Fever or diaphoresis.	Values for BC parameters were derived from the study.
Cornejo-Pareja et al. (18)	Single-frequency 50 kHz, phase-sensitive impedance analyzer—BIA 101 Whole Body Bioimpedance Vector Analyzer (AKERN, Italy).	Within 72 h after admission.	Once	Not reported	PhA, SPhA, BIVA, and hydration status.	Protocol reported. Values extracted directly from the device.	Extensive skin lesions, hematomas, ethnicity, and extravasation of fluids, among others.	Values for BC parameters were derived from the study.
Da Porto et al. (19)	Fixed frequency device, SECA® (model mBCA 525; Seca gmbh and Co, Hamburg, Germany).	Within 36 h after admission.	Once	Not reported	EBW, TBW, FFM, MM, VAT, PhA, BIVA, and EBW/TBW ratio.	Protocol for BIA reported. Values extracted directly from the device, and then transformed.	Pregnancy, anasarca, presence of pacemakers, arthroplasty, or active ECG monitoring, patients with limb amputations or any other reason that impaired the placement of the electrodes and palliative care.	Values for BC were derived from the study.
Kellnar et al. (20)	Nutribox body impedance analyzer (Data Input, Germany).	Within 24 h after admission.	Performed again on day 3 ± 1, and on the day of discharge.	Not reported	Body water, PhA, FM, BCM, and ECM.	Protocol for BIA reported. Values extracted directly from the device.	Patients eligible for outpatient treatment or admitted to the ICU.	Values for BC parameters were derived from the study.
Moonen et al. (21)	InBody S10® (InBody Co., Ltd., Seoul, Korea).Multi-frequency BIA.	Not stated	Once	Trained researcher	TBW, EBW, PhA, FFM, FM, LM, VAT area, SMI, and SLM.	Protocol reported. Values extracted directly from the device, and from a software.	Presence of electrical implants (e.g., pacemakers) pregnancy, wounds or other damage at the designated electrode sites, or incapability to maintain posture during the assessment.	Predetermined reference values for adequate BC parameters were provided.
Moonen et al. (22)	InBody S10® (InBody Co., Ltd., Seoul, Korea). Multi-frequency BIA.	Within 24 h after admission.	Once	Trained researchers	Mineral mass, bone mineral content, VAT area, MM, FFM, SLM, % of body fat, BCM, SMI, ECW/TBW ratio, protein mass, and PhA.	Protocol for BIA reported. Values extracted directly from the device, and from software.	Same as the exclusion criteria mentioned above in the study by Moonen et al. (21)^*^.	Predetermined reference values for adequate BC parameters were provided.
							^*^Presence of electrical implants (e.g., pacemakers) pregnancy, wounds or other damage at the designated electrode sites, or incapability to maintain posture during the assessment	
Cornejo-Pareja et al. (18)	Single-frequency 50 kHz, phase-sensitive impedance analyzer—BIA 101 Whole Body Bioimpedance Vector Analyzer (AKERN, Italy).	Within 72 h after admission.	Once	Not reported	PhA, SPhA, BIVA, and hydration status.	Protocol reported. Values extracted directly from the device.	Extensive skin lesions, hematomas, ethnicity, and extravasation of fluids, among others.	Values for BC parameters were derived from the study.
Hegde et al. (24)	BIA measured at four frequencies. (BIA, Quadscan 4000, Bodystat Ltd., British Isles).	Upon admission	Once	Not reported	FM.	Protocol for BIA reported. Values extracted from and calculated based on the equations provided by the manufacturer.	Pregnancy, admission in ICU, on inotropic support, dialysis, or inability to maintain posture during the assessment.	Values for BC parameters were derived from the study.
Moonen et al. (9)	InBody S10® (InBody Co., Ltd., Seoul, Korea).	Within 24 h after hospital admission	Once	Trained researchers	TBW and LM.	Protocol for BIA reported. Values extracted directly from the device.	Electrical implants, inability to maintain posture for 5 min, pregnancy, or presence of wounds or skin damage at the designated electrode sites.	Values for BC parameters were derived from the study.
Osuna-Padilla et al. (25)	InBody S10® (InBody Co., Ltd., Seoul, Korea). Multifrequency BIA.	Within 48 h after admission at ICU.	Once	ICU dietitian.	PhA, SPA, ECW/TBW ratio, TBW, ICW, and ECW.	Protocol for BIA reported. Values extracted directly from the device, and then transformed.	Not reported	Values for BC parameters were derived from the study.
Reyes-Torres et al. (26)	InBody S10 (InBody Co, Ltd., Seoul, Korea).	After extubation.	Once	Healthcare professional	PhA, TBW, ECW, and ECW/TBW ratio	Protocol for BIA reported. Values extracted directly from the device.	Patients who could not be weighed.	Cutoff values for BC parameters were reported.
Ryrsø et al. (27)	BioScan touch i8 (Maltron International Ltd., United Kingdom)	Within the first 48 h	Once	Not reported	FFM and FM.	Protocol for BIA not reported. Values extracted directly from the device.	Patients with no pathogen detection.	Values for BC parameters were derived from the study.
Stevanovic et al. (33)	TANITA BC-543 apparatus (Tanita Corporation, Tokyo, Japan)	Within the first 72 h of admission.	Once	Not reported	VAT and % of FM.	Protocol for BIA reported. Authors did not specify if the values were derived from the device or software.	Hospitalization due to other reasons than COVID-19, pregnancy, postpartum period, or impossibility to perform anthropometric measurements.	Values for BC parameters were derived from the study.
Andrade-Júnior et al. (28)	B-mode (Logiq e ultrasound, GE Healthcare, United States).	Upon admission and on day 10 of hospital stay.	Twice (day 1 and day 10).	Not reported	MM loss was assessed by means of US. RF CSA (cm2) and the thickness of the anterior compartment of the quadriceps muscle (rectus femoris and vastus intermedius) (cm).	Protocol for US measurement not reported.	Cardiorespiratory instability during the evaluation.	Values for BC parameters were derived from the study.
Bologna and Pone (29)	Model not specified.	Date of admission and at discharge or transfer to another care unit.	Twice	US technician	VLat muscle thickness.	Protocol for US measurement reported.	Need for MV, severe hepatic and renal impairment, severe heart disease, dementia, highly probable death within 24 h, edemas, myositis, anasarca, or use of corticosteroids among others.	Values for BC parameters were derived from the study.
Formenti et al. (30)	B-mode 6 to14 MHz linear array on a Mindray TE-7 machine (Shenzhen Mindray Bio-Medical Electronics Co. Ltd. Shenzen, China)	Within 24 h after admission at ICU.	Once	Single experienced operator.	The thickness (cm) and echogenicity (AU) of the right intercostal, left intercostal, diaphragm, and rectus femoris as well as rectus femoris area (cm^2^).	Protocol for US measurement reported.	Age < 18 years, history of severe chronic obstructive pulmonary disease, pregnancy, or inability to perform respiratory muscle US.	Values for BC parameters were derived from the study.
Gil et al. (31)	B‐mode ultrasound with a 7.5‐MHz linear‐array probe (SonoAce R3, Samsung‐Medison, Gangwon‐do, South Korea)	Within <48 h upon hospital admission.	Once	Performed by a single investigator.	VLat CSA.	Protocol for US measurement not reported.	Neoplasia in the past 5 years, cognitive deficit, delirium, diagnosis of muscle degenerative diseases, or prior admission to invasive MV.	Values for BC parameters were derived from the study.
Umbrello et al. (6)	B-mode −6 to 14 MHz linear array on a Esaote MyLab X8 device (Esaote SpA, Genova, Italy).	Within 24 h after admission at ICU.	Once or twice (repeated at day 7).	Single, experienced operator and reviewed by a second investigator.	RF CSA, echodensity (AU), and thickness (mm) of the diaphragm and rectus femoris.	Protocol for US measurement reported.	Age < 18 years, trauma to the right lower limb, pregnancy, history of neuromuscular, neurologic, or muscular wasting disease, and prolonged immobility before admission to the ICU.	Values for BC parameters were derived from the study.
Kremer et al. (32)	Aplico i800 ultrasound system (Canon, Tokyo Japan) or ACUSON Freestyle ultrasound system (Siemens Healthcare, Erlangen, Germany).	Within 48 h upon hospital admission.	Once	Not reported	Compressed tight muscles thickness index, TMThic, psoas muscle thickness index (PMTI), PMAI, PMA.	Protocol for US measurement reported.	Degenerative muscular diseases.	A non-COVID-19 cohort was used as reference to compare the findings of BC.
Battisti et al. (34)	CT at the level of the second lumbar vertebra.	Upon emergency department admission	Once	Not reported	Abdominal SAT, VAT, and VAT to SAT ratio.	Protocol for CT measurement partially reported. Protocol for BC analysis partially reported.	Unavailability of RT-PCR data or absence of HR-CT signs of pneumonia.	A non-COVID-19 cohort was used as reference to compare the findings of BC.
Favre et al. (35)	CT at the level between the third and the fourth lumbar vertebrae.	Not reported	Once	Performed by a radiologist.	VAT area, SAT area, and VAT/SAT areas ratio.	Protocol for CT measurement not reported. Protocol for BC analysis not reported.	Not reported	Values for BC parameters were derived from the study.
Gualtieri et al. (36)	CT at the level of the 12th thoracic vertebra.	Within 24 h upon admission at ICU and about 20 days later.	Twice	Two different operators. In case of disagreement, a third operator was asked to repeat the analysis.	FM, erector spinae muscle area, and attenuation.	Protocol for CT measurement partially reported. Protocol for BC analysis reported.	Acquired immunodeficiency, history of neutropenia, prior transplant operations, incomplete data, history of immunosuppressive therapy, or absence of the CT scan for the second evaluation.	Patients were classified as lean and obese, according to % of FM.
Kottlors et al. (37)	CT at the level of the 12th thoracic vertebra.	Not reported.	Once	Radiologist	Circumferences of waist and muscle through CT and adipose tissue/muscle ratio.	Protocol for CT measurement reported. Protocol for BC analysis reported.	Other acute pathology, <18 years old.	Values for BC parameters were derived from the study.
Petersen et al. (38)	CT at the middle of the first lumbar vertebra level.	Upon admission—no time specified.	Once	Not reported.	TAT area,SAT, and VAT areas.	Protocol for CT measurement reported. Protocol for BC analysis partially reported.	All CT datasets were of diagnostic image quality and none of the patients had to be excluded.	Values for BC parameters were derived from the study.
Watanabe et al. (39)	CT at the level of the first slice where the bases of the lungs were no longer visible.	Not reported	Once	Two radiologists in consensus.	TAT, VAT, and SAT	Protocol for CT measurement reported. Protocol for BC analysis reported.	Motion artifact, other technical issues that impaired the field of view for adipose tissue, CT acquired with contrast medium, patients without outcomes data.	Values for BC parameters were derived from the study.
Yang et al. (40)	CT at the level of the third lumbar vertebra.	CT scan less than 2 weeks prior to the onset of symptoms.	Once	Two radiologists.	VAT, VAT to SAT ratio, SAT, IMF, and SMA.	Protocol for CT measurement reported. Protocol for BC analysis reported.	Abdominal CT scan 2 weeks prior to the onset of symptoms, contrast-enhanced CT of the abdomen, suboptimal image quality for analysis due to artifacts or ascites, insufficient scanning coverage for imaging evaluation for SAT, and patients who died of causes other than COVID-19.	Predetermined reference values for adequate BC parameters were provided.
Besutti et al. (41)	CT at the level of the seventh and eighth thoracic vertebrae.	Not reported	Once	A single trained analyzer under the supervision of a senior radiologist.	TAT, SAT, VAT, and IMAT areas.	Protocol for CT measurement reported. Protocol for BC analysis reported.	CT scans with artifacts due to pacemakers, with a small field of view, or patients with thoracic lipomas.	Values for BC parameters were derived from the study.
Bunnell et al. (42)	CT at the level of the fourth lumbar vertebra.	Within 2 months of hospital admission	Once	One trained observer.	SAT, VAT, IMAT, and abdominal and paraspinal muscle.	Protocol for CT measurement reported. Protocol for BC analysis reported.	Patients who did not undergo CT scans.	Values for BC parameters were derived from the study.
Chandarana et al. (15)	CT scans at the level of the third lumbar vertebra.	During the acute presentation of SARS-CoV-2 infection or prior abdominopelvic CT with 6 months of the diagnosis of SARS-CoV-2 infection	Once	Two readers	VAT, SAT, IMAT, MI, and MM and ratios of IMAT/MM and VAT/TAT.	Protocol for CT measurement reported. Protocol for BC analysis reported.	Patients whose CT exams showed poor image quality and extensive ascites.	Values for BC parameters were derived from the study.
Chandarana et al. (16)	CT at the level of the third lumbar vertebra.	Not reported	Once	Manually performed by a reader.	SAT, VAT, TAT, and VAT/TAT ratio.	Protocol for CT measurement not reported. Protocol for BC analysis reported.	COVID-19 positivity cutoff not confirmed, unavailability of axial imaging for analysis, and presence of extensive ascites.	Values for BC parameters were derived from the study.
Damanti et al. (43)	CT at the level of the first, second, and third lumbar vertebra (L3 were preferably chosen when available).	Not reported	Once	Two trained radiology residents supervised by a senior radiologist.	Cross sectional areas of abdominal adipose tissue and MM, and SMI.	Protocol for CT measurement reported. Protocol for BC analysis reported.	Low CT scan quality, presence or artifacts, lack of lumbar vertebrae scan images, no treatment with MV.	Values for BC parameters were derived from the study; however, it utilized cutoff values from another for muscle attenuation.
Feng et al. (44)	CT at level of the 12th thoracic vertebrae.	Upon admission. Recovered patients had a second measurement 1 month after discharge.	Once or twice	Two experienced radiologists.	PrMA^*^, PrMD^*^, and PrMI.	Protocol for CT measurement reported. Protocol for BC analysis reported.	CT scan without the 12th thoracic (T12) vertebra scan (n = 12) and lack of complete clinical or laboratory data.	Values for BC parameters were derived from the study.
Giraudo et al. (7)	CT at the level of the 12th thoracic vertebra.	First 3 weeks of hospitalization.	Once	One radiologist.	Reduced MM (HU < 30) at the paravertebral muscle.	Protocol for CT measurement partially reported. Protocol for BC analysis reported.	CT scan performed only after 3 weeks of hospital admission, children, or exam performed only with a contrast enhanced CT.	Predetermined reference values for adequate BC parameters were provided.
Goehler et al. (45)	CT at the level of the first lumbar vertebra.	Within a median (IQR) of 17 (4–25) months before the hospitalization date.	Once	Artificial Intelligence	VAT and SAT.	Protocol for CT measurement partially reported. Protocol for BC analysis reported.	The presence of active malignancy	Predetermined reference values for adequate BC parameters were provided.
Hoyois et al. (46)	CT at the level of the chest scans for dorsal muscle area assessment.	Upon admission.	Once or twice	Evaluator not reported.	DMI.	Protocol for CT measurement reported on supplementary material. Protocol for BC analysis reported.	Not reported.	Values for BC parameters were derived from the study.
McGovern et al. (47)	CT at the third lumbar vertebrae level.	Within 3 months of their positive RT-PCR test for COVID-19.	Once	Automated tool. ImageJ (NIH ImageJ version 1.47).	TAT, VAT, and SMA.	Protocol for CT measurement not reported. Protocol for BC analysis reported.	Lack of scans of the third lumbar vertebra, CT scan older than 3 months, significant movement artifact on the CT scan.	Predetermined reference values for adequate BC parameters were provided.
Moctezuma-Velázquez et al. (48)	CT at the level of the 12th thoracic vertebra.	Upon admission (no time limit specified).	Once	Two trained observers.	SMA, SMI, and MM.	Protocol for CT measurement reported. Protocol for BC analysis reported.	Patients without CT.	Predetermined reference values for adequate BC parameters were provided.
Nobel et al. (49)	CT at the level of the third lumbar vertebra.	Within 30 days before or after the SARS-CoV-2 test.	Once	Analyzed by a single reader.	MM area, VAT, SAT area, and IMAT.	Protocol for CT measurement reported. Protocol for BC analysis reported.	Lack of CT scans within 30 days of the COVID-19 test.	Values for BC parameters were derived from the study.
Ogata et al. (50)	CT at the level of the upper pole of the right kidney.	Not stated	Once	A radiologic technologist.	VAT, SAT, TAT, and VAT/TAT.	Protocol for CT measurement reported. Protocol for BC analysis reported.	No available medical records for follow-up, admission later than 14 days after the onset of the disease, severe state upon admission, lack of CT scans.	Values for BC parameters were derived from the study.
Pediconi et al. (51)	CT at the level of the third lumbar vertebra.	No time limitation was set.First—upon admissionSecond—during hospitalization	Twice	Two radiologists	VAT and SAT areas.	Protocol for CT measurement reported. Protocol for BC analysis reported.	Not reported.	Predetermined reference values for adequate BC parameters were provided.
Polat et al. (52)	CT at the second lumbar vertebra.	Upon hospital admission.	Once	Performed independently and manually by a radiologist and a physiatrist.	Psoas CSA, PMI, and PMD.	Protocol for CT not measurement reported. Protocol for BC analysis not reported.	History of spinal surgery, history of ICU stay for any reason, artifacts on CT scans, or presence of scoliosis.	Values for BC parameters were derived from the study.
Poros et al. (53)	CT at the level of the fifth thoracic vertebrae and between the first lumbar vertebra and 12th thoracic vertebra levels.	Obtained before or shortly after intubation.	Once	One experienced investigator.	The total MM area (at the level of the fifth thoracic vertebra) and VAT (at the level of the between the T12 and L1 vertebra levels).	Protocol for CT measurement partially reported. Protocol for BC analysis reported.	Palliative care, death due to causes other than COVID-19.	Values for BC parameters were derived from the study.
Rossi et al. (54)	CT at the level of the third and fourth lumbar vertebrae.	Not reported	Once	Two trained operators.	SMD and IMAT.	Protocol for CT measurement not reported. Protocol for BC analysis partially reported.	Not reported	Values for BC parameters were derived from the study.
Scheffler et al. (55)	CT at the level of the first abdominal slice caudal to the deepest pleural recess level.	Not reported	Not reported	One trained radiologic technologist.	TAT, SAT, and VAT.	Protocol for CT measurement reported. Protocol for BC analysis reported.	The only exclusion criterion was refusal to participate in a research study	Values for BC parameters were derived from the study.
Schiaffino et al. (56)	CT at the level of the 5th and 12th thoracic vertebrae.	Within 24 h upon hospital admission.	Once	Performed by four radiologists.	Paravertebral muscle areas.	Protocol for CT measurement reported. Protocol for BC analysis partially reported.	Presence of diseases that chronically impair muscular status, inadequate CT image quality, impairment of the adequate segmentation of the paravertebral SMA.	Values for BC parameters were derived from the study.
Viddeleer et al. (57)	CT at the level of the 12th thoracic vertebra in the slice showing both transverse processes.	Upon admission (no time limit specified).	Once	Experienced operator.	SMA, SMI, SAT, mean radiodensity, and IMAT.	Protocol for CT measurement reported. Protocol for BC analysis reported.	Not reported	Values for BC parameters were derived from the study.
Antonarelli and Fogante. (58)	CT at the level of the thorax.	7 days before the intubation.	Once	Two trained radiologists	PMA and PMD derived from chest CT scans (fourth thoracic vertebra) were evaluated.	Protocol for CT measurement reported. Protocol for BC analysis reported.	Artifacts on scans, <18 years old, and impossibility to assess pectoral muscles due to the field of view on chest CT scans.	Values for BC parameters were derived from the study.
Attaway et al. (59)	CT at the level of the 12th thoracic vertebrae.	The interval between the two scans should be at least 3 days.	Twice	Experienced investigator	Pectoralis muscle and erector spinae muscle areas.	Protocol for CT measurement not reported. Protocol for BC analysis reported.	Patients without COVID-19, <18 years, not hospitalized and lacking two CT scans of the chest with at least 3 days interval between them.	Values for BC parameters were derived from the study.
Beltrão et al. (60)	CT at the level of the thorax (thoracoabdominal scan between T12 and L2).	Not reported	Once	Experienced radiologist	VAT, SAT, MA, VAT/SAT ratio, and VAT/MA ratio.	Protocol for CT measurement reported. Protocol for BC analysis reported.	Pregnancy, history of thyroid disease, patients who used iodinated contrast in the last 6 months or other medications affecting thyroid metabolism.	Values for BC parameters were derived from the study.
Bodolea et al. (61)	CT at the level between the seventh and eighth thoracic vertebrae.	Within 24 h upon admission.	Once	A single radiologist has reinterpreted all thoracic CT scans.	SAT, TAT, PMA, and PMD.	Protocol for CT measurement reported. Protocol for BC analysis reported.	Patients without complete laboratory work-up or poor-quality CT scans that reduced the adequate assessment.	Values for BC parameters were derived from the study.
Do Amaral e Castro et al. (62)	CT at level of the thorax.	Within 24 h upon admission.	Once	Not reported	SAT and pectoral and paravertebral MM.	Protocol for CT measurement reported. Protocol for BC analysis reported.	Not reported.Supplementary material not found.	Values for BC parameters were derived from the study.
Faiella et al. (63)	CT at the level of the third lumbar vertebra.	During hospitalization.	Once	Not reported^a^.	VAT, SAT, and VAT/SAT.	Protocol for CT measurement reported. Protocol for BC analysis reported	Patients < 18 years of age and non-COVID-19 patients.	Values for BC parameters were derived from the study.
				^a^Image processor described image processing application (OsiriX, Pixmeo, Bernex, Switzerland)				
McGovern et al. (64)	CT at the third lumbar vertebrae level.	Within 3 months of their positive RT-PCR test for COVID-19.	Once	Automated tool. ImageJ (NIH ImageJ version 1.47).	TAT, VAT, and SMA.	Protocol for CT measurement not reported. Protocol for BC analysis reported.	Lack of scans of the third lumbar vertebra, CT scan older than 3 months, significant movement artifact on the CT scan.	Predetermined reference values for adequate BC parameters were provided.
Menozzi et al. (65)	CT at the 12th thoracic vertebrae level.	During hospitalization.	Once	Automated tool—not specified.	SMA and sarcopenia.	Protocol for CT measurement reported. Protocol for BC analysis not reported.	Lack of available CT scan.	Predetermined reference values for adequate BC parameters were provided.
Molwitz et al. (66)	CT at the 12th thoracic and 3rd lumbar vertebra levels.	The first scan during hospitalization.	Once	Automated tool. ImageJ (National Institutes of Health and the Laboratory for Optical and Computational Instrumentation, USA).	SMA, SMI, MRA, SAT, and VAT from L3 and T12.	Protocol for CT measurement reported. Protocol for BC analysis not reported.	Artifacts in the paravertebral muscle, CT done without the whole abdominal muscle area, display of an open abdomen.	Predetermined reference ranges based on ideal BC were provided. SMI and MRA had predetermined reference ranges.

BC, body composition; BIA, bioelectrical impedance; BIVA, bioelectrical impedance vector analysis; CT, computed tomography; CSA, cross-sectional area; DMI, height normalized index of dorsal muscle area; ICU, intensive care unit; MV, mechanical ventilation; ECW, extracellular water; ECW/TBW, extracellular water/total body water; ECM, extracellular mass; LM, lean mass or SLM soft lean mass; MAT/MM, muscular adipose tissue/muscle mass; MM, muscle mass; MRA, muscle radiodensity attenuation; PhA, phase angle; PMT, psoas muscle thickness; PMA, psoas muscle area; PMD, psoas muscle density; PMAI, psoas muscle area index; PrMA, paraspinal muscle area; PrMD, paraspinal muscle density; PrMI^*^, paraspinal muscle index; NRS 2002, Nutrition Risk Screening 2002; SAT, subcutaneous adipose tissue; SMA, skeletal muscle area; SMI, skeletal mass index; TAT, total adipose tissue; TBW, total body water; TMThic, tight muscles thickness; VAT, visceral adipose tissue; VAT/SAT, visceral adipose tissue/subcutaneous adipose tissue.

**Table 3:** Findings of body composition of included studies.

Reference	Assessment tool and results of the total sample															
	Bioelectrical impedance	PhA (º)	SPhA	BCM	ECM	TBW	FM	FFM	FFMI	SLM or LM^*^	VAT	ICW	ECW	SMI	MM	ECW/TBW
Del Giorno et al. (17)	All patients = 90	5.6 ± 1.14	N/A	17.8 ± 4.7 (kg/m)	N/A	25.5 ± 4.2 (L/m)	21.1 ± 9.1 (kg)	58.2 ± 10.7 (kg)	34.3 ± 6.0 (kg/m)	N/A	N/A	N/A	N/A	N/A	N/A	N/A
	Mean ± SD															
Cornejo-Pareja et al. (18)	All patients = 127	4.4 (3.2, 5.4)	−0.8 (−2.0, − 0.2)	21.4 (16.3, 27.9) (kg)	N/A	N/A	N/A	N/A	N/A	N/A	N/A	N/A	N/A	N/A	N/A	N/A
	Median (IQR)															
Da Porto et al. (19)	All patients = 150	5.5 ± 1.5	N/A	N/A	N/A	44.6 ± 10.1	28.8 ± 10.1 (kg/m^2^)	59.1 ± 13.3 (kg/m^2^)	N/A	N/A	3.4 ± 2.1 (L)	N/A	12.2 ± 3.9	N/A	N/A	45.1 ± 3.3
	Mean ± SD															
Kellnar et al. (20)	All patients = 12	5.6	N/A	50.2 (44.1–55.1) (%)	49.8 (44.9–56.0) (%)	51.0 (38.5–54.6) (%)	30.1(24.9–32.1) (%)	N/A	N/A	N/A	N/A	N/A	N/A	N/A	N/A	N/A
	Median (IQR)															
Moonen et al. (21)	All patients = 54	4.5 (4.2–4.8)	N/A	N/A	N/A	44.7 (41.8–47.6) (L)	29.7 (25.9–33.6) (kg)	59.2 (55.4–63.1) (kg)	N/A	55.9 (52.3–59.5) (kg)	155.2 (136.1–174.2) (cm^2^)	26.9 (25.2–28.7) (L)	17.8 (16.6–18.9) (L)	8.0 (7.6–8.4) (kg/m^2^)	N/A	0.40 (0.39–0.40) (L)
	Median (IQR)															
Moonen et al. (22)	All patients = 150	5.4 (5.2–5.6)	N/A	37.7 (36.2–39.2) (kg)	N/A	42.9 (41.4–44.6) (L)	30.1 (27.9–32.3) (kg)	58.5 (56.3–60.7) (kg)	N/A	55.1 (53.1–57.2) (kg)	154 (144–166) (cm^2^)	26.2 (25.3–27.4) (L)	16.7 (16.2–17.3) (L)	8.1 (7.8–8.3) (kg/m^2^)	N/A	0.39 (0.39–0.39)
	Median (IQR)															
Cornejo-Pareja et al. (23)	All patients = 127	N/A	N/A	N/A	N/A	N/A	N/A	N/A	N/A	N/A	N/A	N/A	N/A	N/A	N/A	0.55 (0.49–0.63)
	Median (IQR)															
Hegde et al. (24)	All patients = 172	N/A	N/A	N/A	N/A	N/A	33.4 ± 9.4 and 30.5 (25.9, 40.3) (%)	N/A	N/A	N/A	N/A	N/A	N/A	N/A	N/A	N/A
	Mean ± SD and median (IQR)															
Moonen et al. (9)	All patients = 150	N/A	N/A	N/A	N/A	88 (85.0–91.0)	N/A	N/A	N/A	58.5 (56.3–60.7) (kg) ^*^LM	N/A	N/A	N/A	N/A	N/A	N/A
	Median (IQR)															
Osuna-Padilla et al. (25)	All patients = 67	5.0 ± 1.2	−2.5 (−3.8, −0.83)	N/A	N/A	40.8 ± 7.5 (L)	N/A	N/A	N/A	N/A	N/A	24.9 ± 4.8 (L)	15.9 ± 2.8 (L)	N/A	N/A	0.39 ± 0.01
	Mean ± SD															
Reyes-Torres et al. (26)	All patients = 112	4.8 ± 1.1	N/A	N/A	N/A	N/A	N/A	N/A	N/A	N/A	N/A	N/A	N/A	N/A	N/A	0.395 ± 0.138
	Mean ± SD															
Ryrsø et al. (27)	All patients = 40	N/A	N/A	N/A	N/A	N/A	27.7 ± 8.0 (%)	55.1 ± 13.7 (kg)	N/A	N/A	N/A	N/A	N/A	N/A	N/A	N/A
	Mean ± SD															
Stevanovic et al. (33)	All patients = 216	N/A	N/A	N/A	N/A	N/A	50.9%	N/A	N/A	N/A	N/A	Very high level 38.4%	N/A	N/A	N/A	N/A
	Frequency (%)															
	Ultrasound	VLat	VLatEcho	VInt	VIntEcho	TMThic	PMT	PMA	PMAindex	RFThic	RFA	RF Echo	DiaP Thic	DiaP Echo	ACQ	TightThicIndex
Andrade-Júnior et al. (28)	All patients = 32	N/A	N/A	N/A	N/A	N/A	N/A	N/A	N/A	N/A	−30.1%	N/A	N/A	N/A	−18.6%	N/A
	Decrease in %															
Bologna and Pone (29)	All patients = 80MeanTreatment vs. no treatment	2.18 to 2.06 vs. 2.23 to 1.88	N/A	N/A	N/A	N/A	N/A	N/A	N/A	N/A	N/A	N/A	N/A	N/A	N/A	N/A
Formenti et al. (30)	All patients = 32	N/A	N/A	N/A	N/A	N/A	N/A	N/A	N/A	0.59 (0.56–0.69) (cm)	1.83 (1.2–2.6) (cm^2^)	N/A	0.25 (0.19–0.28) (cm)	74.1 (65.1–84.0) (AU)	N/A	N/A
	Median (IQR)															
Gil et al. (31)	All patients = 186	12 (12–19) (cm^3^)	N/A	N/A	N/A	N/A	N/A	N/A	N/A	N/A	N/A	N/A	N/A	N/A	N/A	N/A
	Median (IQR)															
Umbrello et al. (6)	All patients = 28	N/A	N/A	N/A	N/A	N/A	N/A	N/A	N/A	N/A	2.98 (2.17–3.97)vs. 2.49 (2.04–3.34) (cm^2^)	84.9 (75.9–94.9) vs. 90.6 (82.9–102.2) (AU)	2.21 (1.87–2.58) vs. 2.14 (1.95–3.00) (cm)	75.1 (66.8–84.4) vs. 93.7 (82.2–97.9) (AU)	N/A	N/A
	Median (IQR)Survivors vs. non-survivors															
Kremer et al. (32)	All patients = 113	N/A	N/A	N/A	N/A	24.5 (20.0–32.0) (mm)	30.5 (26.2–37.0) (mm)	730.8 (5,435–1,078.4) (mm^2^)	251.5 (190.1–353.7) (mm^2^/m^2^)	N/A	N/A	N/A	N/A	N/A	N/A	14.6 (11.5–19.2) (mm/m)
	Median (IQR)															
	Computed tomography	VAT	SAT	TAT	IMAT	IMT	VAT/SAT	DMI	SMI	MM	SMD	PM	ESMa	ESMat	VAT/TAT	PEC
Battisti et al. (34)	All patients = 144	15.1 ± 6.6 (mm)	17.7 ± 8.9 (mm)	N/A	N/A	N/A	1.16 ± 0.93	N/A	N/A	N/A	N/A	N/A	N/A	N/A	N/A	N/A
	Mean ± SD															
Favre et al. (35)	All patients = 165	131.7 ± 101.3 (cm^2^)	152.8 ± 103.4 (cm^2^)	N/A	N/A	N/A	N/A	N/A	N/A	N/A	N/A	N/A	N/A	N/A	N/A	N/A
	Mean ± SD															
Gualtieri et al. (36)	All patients = 150	N/A	N/A	N/A	N/A	N/A	N/A	N/A	N/A	N/A	N/A	N/A	20.08 ± 4.52 (cm^2^)	27.63 ± 3.24 (HU)	N/A	N/A
	Mean ± SD															
Kottlors et al. (37)	All patients = 58	FMR = 5.9 ± 1.3	N/A	N/A	N/A	N/A	N/A	N/A	N/A	N/A	N/A	N/A	N/A	N/A	N/A	N/A
	Mean ± SD															
Petersen et al. (38)	All patients = 30	8.2 (5.5) 10 cm^2^	6.2 (4.8) 10 cm^2^	15.1 (7.6) 10 cm^2^	N/A	N/A	N/A	N/A	N/A	N/A	N/A	N/A	N/A	N/A	N/A	N/A
	Median (IQR)															
Watanabe et al. (39)	All patients = 215	14,331.51 ± 8,372.32 (mm^2^)	13,745.39 ± 8,506.76 (mm^2^)	28,076.90 ± 14,016.29 (mm^2^)	N/A	N/A	N/A	N/A	N/A	N/A	N/A	N/A	N/A	N/A	N/A	N/A
Yang et al. (40)	All patients = 143	103.4 (60.3–166.6) (cm^2^)	108.2 (77.0–156.7) (cm^2^)	N/A	N/A	N/A	N/A	N/A	N/A	96.2 (79.0–118.2) (cm^2^)	32.3 (23.7–39.3) (HU)	N/A	N/A	N/A	N/A	N/A
	Median (IQR)															
Besutti et al. (41)	All patients = 318	152 (102.0–210.0) (cm^2^)	27.0 (18.0–37.0) (cm^2^)	34.0 (23.0–47.0) (cm^2^)	223.5 (159.0–292.5) (cm^2^)	N/A	N/A	N/A	N/A	N/A	^*^34 (27–41) HU	N/A	N/A	N/A	N/A	17 (12–21)cm^2^
	Median (IQR)															
Bunnell et al. (42)	All patients = 124	145.6 (86.2–210.9) (cm^2^)	269.9 (198.1–386.6) (cm^2^)	N/A	12.1 (6.1–20.7) (cm^2^)	N/A	0.51 (0.29, 0.83)	N/A	N/A	134.5 (113.4–167.3) (cm^2^)	N/A	N/A	N/A	N/A	N/A	N/A
	Median (IQR)															
Chandarana et al. (15)	All patients = 177Mean ± SD and Median (IQR)Inpatients vs. outpatients	234.8 ± 112.1 vs. 157.9 ± 92.4 (cm^2^)	N/A	N/A	17.8 ± 9.4 vs. 12.1 ± 7.0 (cm^2^)	N/A	N/A	N/A	N/A	122.7 ± 34.5 vs. 131.0 ± 32.8 (cm^2^)	N/A	N/A	N/A	N/A	0.48 ± 0.14 vs. 0.38 ± 0.16	N/A
Chandarana et al. (16)	All patients = 30	228.6 ± 111.1 vs. 128.0 ± 92.1 (cm^2^)	N/A	N/A	N/A	N/A	N/A	N/A	N/A	N/A	N/A	N/A	N/A	N/A	0.52 ± 0.14 vs. 0.35 ± 0.20	N/A
	Mean ± SD															
	Hospitalized vs. outpatient															
Damanti et al. (43)	All patients = 81	207.5 ± 87.70 (cm^2^)	164.1 ± 64.54 (cm^2^)	387.2 ± 127.77(cm^2^)	15.6 ± 9.76 (cm^2^)	N/A	N/A	N/A	35.5 (28.9–43.6) (cm^2^/m^2^)	112.9 ± 29.93 (cm^2^)	28.3 ± 8.36 (HU)	N/A	N/A	N/A	N/A	N/A
	Mean ± SD															
Feng et al. (44)	All patients = 116	N/A	N/A	N/A	N/A	N/A	N/A	N/A	^***^11.8 (10.5–13.1) vs. 10.7 (9.8–13.2) (cm^2^/m^2^)	^***^32.5 (25.4–37.5) vs. 28.1 (25.9–34.0) (cm^2^)	^***^41.9 (36.1–47.4) vs. 35.0 (28.1–42.8) (cm^2^/m^2^)	N/A	N/A	N/A	N/A	N/A
	Non-severe COVID-19 vs. severe COVID-19Median (IQR)															
Giraudo et al. (7)	All patients = 150	N/A	N/A	N/A	N/A	N/A	N/A	N/A	N/A	N/A	29 ± 24 vs. 39.4 ± 12 (HU)	N/A	N/A	N/A	N/A	N/A
	Mean ± SD (ICU patients vs. non-ICU)															
Goehler et al. (45)	All patients = 378	195 ± 107 (cm^2^)	N/A	N/A	N/A	N/A	N/A	N/A	N/A	N/A	N/A	N/A	N/A	N/A	N/A	N/A
	Mean ± SD															
Hoyois et al. (46)	All patients = 15	N/A	N/A	N/A	N/A	N/A	N/A	Male 11.8 (11.7–15.9)Female 9.5 (7.3–10.9) (cm^2^/m^2^)	N/A	N/A	N/A	N/A	N/A	N/A	N/A	N/A
	Median (IQR)															
McGovern et al. (47)	All patients = 63	High VAT67.0	High SAT index75.0	N/A	N/A	N/A	N/A	N/A	Low SMI80.2	N/A	Low SMD79.2	N/A	N/A	N/A	N/A	N/A
	Frequency (%)															
Moctezuma-Velázquez et al. (48)	All patients = 519	N/A	N/A	N/A	N/A	N/A	N/A	N/A	44.4 (38.5–51.1) (cm^2^/m^2^)	122.1 ± 29.1 (cm^2^)	N/A	N/A	N/A	N/A	N/A	N/A
	Mean ± SD and Median (IQR)															
Nobel et al. (49)	All patients = 190	Index53.9 (35.1–79.1) vs. 50.1 (27.4–73.5) (cm^2^/m^2^)	Index69.5 (43.2–88.7) vs. 68.0 (49.9–89.8) (cm^2^/m^2^)	N/A	Index2.26 (1.28–4.47) vs. 2.65 (1.72–4.28) (cm^2^/m^2^)	N/A	0.73 (0.43–1.20) vs. 0.65 (0.41–0.94) (cm^2^/m^2^)	N/A	44.9 (35.8) vs. 43.8 (31.5–57.0) (cm^2^/m^2^)	N/A	N/A	N/A	N/A	N/A	N/A	N/A
	Median (IQR)GI symptoms vs. no GI symptoms															
Ogata et al. (50)	All patients = 53	130.7 ± 89.5 (cm^2^)	86.8 ± 51.7 (cm^2^)	217.5 ± 120.2 (cm^2^)	N/A	N/A	N/A	N/A	N/A	N/A	N/A	N/A	N/A	N/A	56.5 ± 19.7 (%)	N/A
	Mean ± SD															
Pediconi et al. (51)	All patients = 62	Non-ICU 154.8 (92.3–256.3) vs. ICU 258.3 (199.5–292.6) (cm^2^)	Non-ICU 170.5 (113.8–234.9) vs. ICU 199.2 (146.9–301.3) (cm^2^)	N/A	N/A	N/A	N/A	N/A	N/A	N/A	N/A	N/A	N/A	N/A	N/A	N/A
Polat et al. (52)	All patients = 130	N/A	N/A	N/A	N/A	N/A	N/A	N/A	0.09 (0.07–0.12) (cm^2^/kg/m^2^)	2.09 (1.55–3.06) (cm^2^)	49.9 (44.6–53.7) (HU)	N/A	N/A	N/A	N/A	N/A
Poros et al. (53)	All patients = 67	125.86 (67.09 N/A164.35) (cm^2^)	N/A	N/A	N/A	N/A	N/A	N/A	N/A	160.78 (133.79–193.79) (cm^2^)	N/A	N/A	N/A	N/A	N/A	39.95 (30.15–49.69) (cm^2^)
	Median (IQR)															
Rossi et al. (54)	All patients = 153	N/A	N/A	N/A	4.36 ± 3.77 (cm^2^) ^*^Psoas	N/A	N/A	N/A	N/A	16.66 ± 9.37 (cm^2^) ^*^Psoas	37.79 ± 8.55 (HU) ^*^Psoas	N/A	N/A	N/A	N/A	N/A
	Mean ± SD															
Scheffler et al. (55)	All patients = 64	141.3 ± 84.0 (mm^2^)	126.2 ± 86.4 (mm^2^)	267.5 ± 143.0 (mm^2^)	N/A	N/A	N/A	N/A	N/A	N/A	N/A	N/A	N/A	N/A	N/A	N/A
	Mean ± SD															
Schiaffino et al. (56)	All patients = 552	N/A	N/A	N/A	N/A	N/A	N/A	T5 6.6 (4.3–11.2)T12 10.8 (8.9–12.8) (cm^2^/m^2^)	N/A	T5 1,940 (1,208–3,189)T12 3,100 (2,499–3,796) (mm^2^)	T5 23 (12–32)T12 37 (24–47) (HU)	N/A	N/A	N/A	N/A	N/A
	Median (IQR) derived from T12															
Viddeleer et al. (57)	All patients = 215	N/A	160.4 (115.7–198.8) vs. 133.1 (97.9–190.6) (cm^2^)	N/A	10.1 (5.0–18.0) vs. 6.2 (3.7–11.4) (cm^2^)	N/A	N/A	N/A	35.7 ± 9.5 vs. 36.1 ± 9.1 (cm^2^/m^2^)	104.0 (83.3–116.7) vs. 108.0 (86.5–124.4) (cm^2^)	24.0 ± 10.1 vs. 27.6 ± 10.9 (HU)	N/A	N/A	N/A	N/A	N/A
	Mean ± SD and median (IQR)Dead vs. alive															
Antonarelli and Fogante (58)	All patients = 112	N/A	N/A	N/A	N/A	N/A	N/A	N/A	N/A	N/A	30.2 ± 6.2 vs. 26.1 ± 4.9^*^	N/A	N/A	N/A	N/A	41.6 ± 8.7 vs. 37.2 ± 6.7 (cm^2^)
	Mean ± SDShorter ICU stay vs. longer ICU stay															
Attaway et al. (59)	All patients = 95	N/A	N/A	N/A	N/A	N/A	N/A	N/A	N/A	N/A	N/A	N/A	37.0 (30.8–46.8) vs. 27.8 (24.5–37.3) (cm^2^)	N/A	N/A	33.1 (26.0–47.0) vs. 31.0 (27.2–34.9) (cm^2^)
	Median (IQR)Alive vs. dead															
Beltrão et al. (60)	All patients = 200	127 (85.8–180.5) (cm^2^)	161.4 (102.4–217.7) (cm^2^)	N/A	N/A	N/A	0.83 (0.54–1.29) (cm^2^)	N/A	N/A	89.6 (75.5–112) (cm^2^)	N/A	N/A	N/A	N/A	N/A	N/A
	Median (IQR)															
Bodolea et al. (61)	All patients = 123	N/A	77.9 (70.9–94.5) (cm^3^)	84.5 (88–105.5) (cm^3^)	N/A	N/A	N/A	N/A	N/A	N/A	N/A	N/A	N/A	N/A	N/A	18.9 (18.1–20.9) (cm^2^)18.5 (16.1–21.2) (HU)
	Median (IQR)															
Do Amaral e Castro et al. (62)	All patients = 123	N/A	Index20.9 ± 13.5 vs. 18.4 ± 11.1 (cm^2^)	61.7 ± 36.8 vs. 54.1 ± 29.2 (cm^2^)	N/A	N/A	N/A	N/A	^**^11.0 ± 10.4 vs. 10.4 ± 2.7 (cm^2^/m^2^)	^**^33.1 ± 8.4 vs. 31.8 ± 10.2	^**^35.1 ± 17.2/ 40.7 ± 13.4	N/A	N/A	N/A	N/A	Index 16.3 ± 18.5 vs. 14.1 ± 5.1 (cm^2^/m^2^)
	Mean ± SDWorse outcome vs. better outcome															
Faiella et al. (63)	All patients = 132	168 ± 84.6 vs. 196 ± 101.9	136 ± 78 vs. 159 ± 82.2	N/A	N/A	N/A	1.5 ± 0.8 vs. 1.5 ± 0.9	N/A	N/A	N/A	N/A	N/A	N/A	N/A	N/A	N/A
	Bleeding group vs. control group															
McGovern et al. (64)	All patients = 106	High	High index	N/A	N/A	N/A	N/A	N/A	Low	N/A	Low	N/A	N/A	N/A	N/A	N/A
	Frequency (%)	67.0	74.5						80.2		79.2					
Menozzi et al. (65)	All patients = 272	N/A	N/A	N/A	N/A	N/A	N/A	N/A	75.0 ± 26.4 vs. 107.6 ± 35.1	N/A	N/A	N/A	N/A	N/A	N/A	N/A
	Mean ± SD															
	First wave group vs. second wave group															
Molwitz et al. (66)	All patients = 46	195.83 ± 111.57 vs. 233.42 ± 101.54	303.26 ± 130.87 vs. 178.66 ± 121.28	N/A	N/A	N/A	N/A	N/A	N/A	96.49 ± 23.23 vs. 125.37 ± 34.99	32.1 ± 11.7 vs. 38.6 ± 12.6	N/A	N/A	N/A	N/A	N/A
	Mean ± SD															
	Female vs. male (L3)															

N/A, Not available; PhA (º), phase angle; SPhA, standardized phase angle; BCM, body cell mass; ECM, extracellular mass; TBW, total body water; FM, fat mass; FFM, fat free mass; FFMI, fat free mass index; SLM or LM, soft lean mass or lean mass; VAT, visceral adipose tissue; ICW, intracellular water; ECW, extracellular water; SMI, skeletal muscle index; ECW/TBW, extracellular water/total body water; VLat, vastus lateralis; VLatEcho, vastus lateralis echogenicity; VInt, vastus intermedius; VIntEcho, vastus intermedius echogenicity; TMThic, thigh muscle thickness; PMT, psoas muscle thickness; PMA, psoas muscle area; PMA, psoas muscle area index; RFThic, rectus femoris thickness; RFA, rectus femoris area; RF Echo, rectus femoris echogenicity; DiaP Thic, diaphragm thickness; DiaP Echo, diaphragm echogenicity; ACQ, anterior compartment of the quadriceps; ThighThicIndex, thigh thickness index; SAT, subcutaneous adipose tissue; TAT, total adipose tissue; IMAT, intermuscular adipose tissue; IMT, intramuscular adipose tissue; VAT/SAT, visceral adipose tissue/subcutaneous adipose tissue; DMI, height normalized index of dorsal muscle area; SMD, skeletal muscle density; PM, paravertebral muscle; ESMa, erector spinae muscle area; ESMat, erector spinae muscle attenuation; VAT/TAT, visceral adipose tissue/total adipose tissue; PEC, pectoralis muscle area/index; ^***^Paraspinal muscle; ^**^Paravertebral muscle; ^*^Pectoralis muscle.

### 2.6. Reporting items

The PRISMA checklist workflow for scoping reviews was used for reporting items in this scoping review (13) and it is available in the Supplementary material 2. No quality appraisal for the studies was performed since it is not recommended for scoping reviews according to JBI (14).

## 3. Results

**Figure 1:**
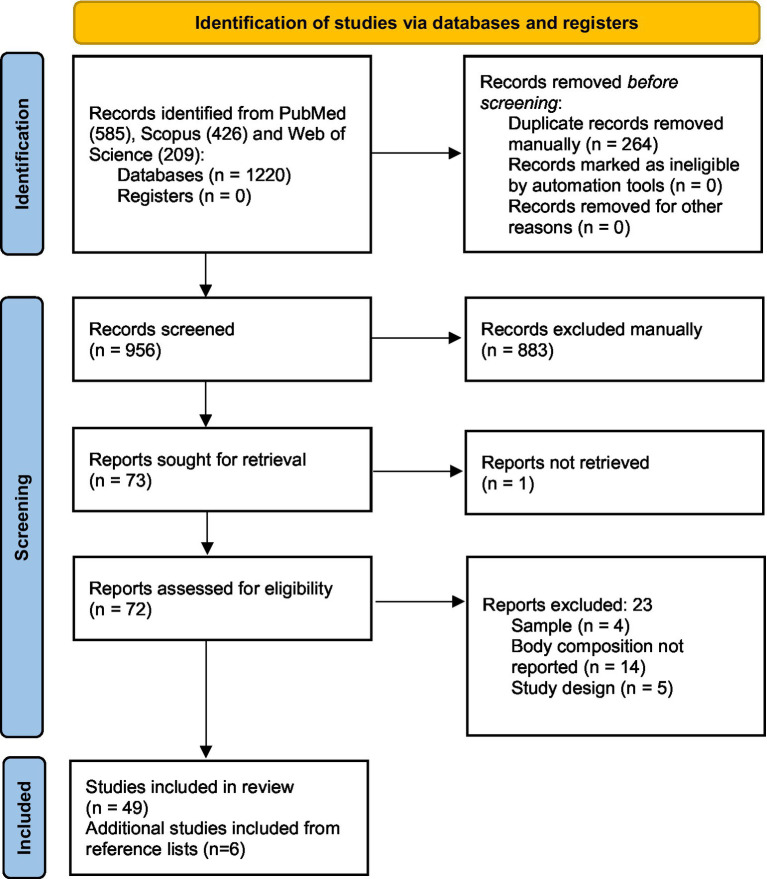
PRISMA flowchart for the study selection.

From the 1,220 potentially relevant citations yielded from the systematic searches, 264 records were excluded due to duplication. After the manual deletion of selected articles, 956 articles were eligible for the title and abstract readings of which 74 were eligible for the full-text assessment. After the full-text selection, 55 studies were eligible for data extraction and inclusion in our study. The flowchart of the study selection is shown in Figure 1.

Concerning the tools utilized, 36 used CT, 13 used BIA, and 6 used US. No studies with D3-creatinine, 24 h urine excretion, DXA, nor MRI for BC assessment were found.

Table 1 presents the studies performing BC assessments in patients hospitalized with COVID-19.

### 3.1. General characteristics of the studies

#### 3.1.1. Year and country of publication

Regarding the year of publication, eight studies were published in 2020 (17, 34–40), 29 in 2021 (6, 7, 15, 16, 18–22, 28, 30, 31, 41–57), and 18 in 2022 (9, 23–27, 29, 32, 33, 58–66). Interestingly, the highest frequency of studies evaluating BC were from the European continent, with 37 studies (67.3%), followed by America with 13 publications (23.6%), Asia with four studies (7.3%), and Eurasia with one study (Table 1).

#### 3.1.2. Study design and objectives

Out of the 55 studies, 40 were single-center (72.7%) (6, 9, 17–23, 25, 27, 28, 30–32, 34, 36, 38, 42, 43, 45–50, 52, 53–55, 58, 60–66) and seven were multicenter cohorts (12.7%) (15, 26, 37, 44, 51, 56, 59); the remaining eight studies did not specify the number of centers (14.6%) (7, 16, 24, 29, 33, 35, 41, 57). Most of the studies (*n* = 46; 83.6%) investigated the associations between abnormal BC markers and changes with COVID-19 outcomes such as ICU admission, disease severity, length of hospital stay (LOS), mechanical ventilation (MV), and death (6, 7, 15–25, 28, 30–34, 36–38, 40–43, 45–49, 50, 52–54, 56–62, 66).

From the studies that did not evaluate BC and COVID-19 outcomes through BIA, one evaluated post-extubation dysphagia (26), one analyzed the agreement of lean mass (LM) between BIA and other measurement tools (9), and one analyzed the BC characteristics between different groups infected by viral and bacterial pathogens (27). Out of the studies with US, one evaluated the treatment and prevention of sarcopenia by arginine supplementation (29). CT on the other hand was used in five studies with different objectives, e.g., assessing nutritional status and outcomes in patients after ICU discharge (46), examining the relationship between patients admitted with COVID-19 and frailty and other prognostic factors (64), investigating obesity or sarcopenia through the T12 and L3 scans (66), and assessing tissue bleeding and BC parameters (63). Finally, Gualtieri, evaluated the differences in BC of aldults with obesity and without obesity during ICU stay (36).

### 3.2. General characteristics of the participants

#### 3.2.1. Sample size

Sample size varied greatly between studies, from 15 participants (46) to 519 (48) in single-center studies, and from 58 (37) to 552 (56) in multicenter studies evaluating BC through CT. When evaluating through BIA, the number of patients enrolled varied from 12 (20) to 216 (33), and for US, it varied from 28 (6) to 186 (31). Out of the included studies, 11 reported the sample size estimation either on the manuscript or in supplementary material (6, 18, 23, 25, 30–32, 36, 39, 43, 60).

#### 3.2.2. Clinical characteristics

Participants evaluated by BIA did not vary greatly between studies. One paper included critically ill patients only (25), Moonen et al. (21) also included ICU patients, and another included post-ICU patients (26). The studies with US focused on the muscular changes of the critically ill (6, 28, 30), and measured the predictive value of one measurement on the prognosis of the moderately to severely ill (31) and non-ICU patients (32). Meanwhile, of the studies that used CT scans, three only evaluated ICU patients (36, 46, 54). In some studies, a cohort of non-COVID-19 patients was enrolled for comparison to the group affected by the disease (32, 34). Regarding the sex of the participants, the most prevalent was male in most of the studies, with frequencies varying from 53.3% (41) to 93.3% (28). A few studies had smaller percentages of males in the sample, the studies of Kremer et al. that had 50% of males (32), McGovern et al. with 47.3% (47), Yang et al. with 46.9% (40), and Faiella et al. (63) with 50% and 46% in the bleeding group and control group, respectively.

The information on the parameters and moment and frequency of evaluation are presented in Table 2.

### 3.3. Body composition methods used in the studies

#### 3.3.1. Body composition assessment tools and parameters assessed

BIA was used in 13 studies (9, 17–27, 33) and the measurements performed differed significantly among them. Some studies only evaluated the parameters of the hydration status, while others evaluated the parameters of fat mass (FM) and LM. In the studies that evaluated BC through BIA, various parameters of hydration and BC were reported. Two studies that utilized the same sample of the ward (*n* = 141) and ICU patients (*n* = 49) relied on predictive equations to assess other BC parameters (9, 22) as well as the study enrolling 54 patients (21). The remaining studies in which ward patients were included explored other BC parameters such as FM and fat-free mass (FFM) Ryrsø et al. (27), Da Porto et al. (19), Del Giorno et al. (17), Stevanovic et al. (33), and Hegde et al. (24) reported data of FM or VAT and not FFM.

Regarding the use of US to evaluate BC, all the studies that assessed either the parameters for MM quantity or quality were reported A total of five studies (5/6, 83.3%) reported the associations between muscle quality and quantity parameters and worse outcomes (6, 28, 30–32). No studies reported data on FM. Additionally, three of the six studies repeated the measurements before discharge or a few days after the first assessment to evaluate MM during hospitalization. In two studies, critically ill patients were included and analyzed through measurements of the diaphragm and rectus femoris echogenicity and thickness (6, 30). The echogenicity, or echodensity, in arbitrary units (AU) was measured in two studies (6, 30).

CT stood out as the most frequently used method to describe BC, with 36 studies (7, 15, 16, 34–37, 39–66). VAT was evaluated in 21 studies (15, 16, 34–36, 39–42, 45, 47, 49–51, 53, 55, 60, 63, 64, 66). MM was evaluated in another 24 studies through muscle quantity (7, 16, 36, 37, 40, 42–44, 48, 49, 52, 53, 56–62, 65, 66), SMD (36, 41, 44, 46, 47, 52, 54, 57, 58, 61, 66), or index (47, 49, 43).

#### 3.3.2. Moment and frequency of evaluation

BIA was mostly evaluated once; Kellnar et al. on the other hand evaluated BC upon admission and on the day of discharge. US was evaluated twice during hospitalization (6, 28, 29) since the objectives were to compare the changes in the muscular tissue during hospitalization. CT, however, was mostly evaluated only once in 30 studies (7, 15, 16, 34, 35, 37–43, 45, 47–50, 52–54, 56–58, 60–66), once or twice in the studies by Hoyois et al. and Feng et al., and twice in the studies by Faiella et al., Pediconi et al., and Gualtieri et al.

Regarding the time of the evaluation, BIA was evaluated mostly within 24 h of admission (9, 17, 20–22) but other authors evaluated it upon 48 h (25, 27) of admission or even 72 h of admission (18, 23, 33). Measurements with US had a narrower interval for the first measurement of a maximum of 24 to 48 h upon admission in the cohorts in Kremer et al., Gi et al., Umbrello et al., and Formenti et al. However, the remaining studies did not provide further information on the moment of evaluation.

The numerical values of the BC parameters of the three tools are presented in Table 3.

### 3.4. Main findings

Some of the studies with BIA found significant results between the parameters derived from the tools and worse prognoses, for instance, phase angle (PhA) (18, 20–22, 25) and percentage of FM (24, 33). Moonen et al. also estimated BC parameters but found only PhA increased the odds of morbidity and mortality in COVID-19 patients (22). The studies utilizing BIA showed mostly PhA to be a strong indicator of severe illness (22), morbidity (21, 22), and mortality according to the studies’ findings (18, 21, 22, 25). Nevertheless, the indicator was not associated with LOS in the studies by Osuna-Padilla et al. (25) and Del Giorno et al. (17). Furthermore, Del Giorno et al. evaluated the associations between BIA parameters and mortality with ICU admission; however, a significant association using the measurements was not found (17).

Regarding the use of US, in three studies, there was an expressive reduction in muscular tissue (6, 28, 29). Furthermore, the reduction of the thickness in the rectus femoris muscle area (28) and vastus lateralis area (31) were predictors of a severe state and LOS, respectively. Higher values of echogenicity of the rectus femoris, diaphragm, and right intercostal sites also showed an association with worse outcomes in the study by Formenti et al. (30) as well as muscle area and thickness (6, 28, 31, 32).

In the studies that aimed to analyze the associations between the VAT and adverse outcomes, many of them found an association between higher values and hospitalization (16, 41), disease severity (35, 45, 55), critical illness (40, 53), MV (41), ICU admission (34, 39, 51), or mortality (41, 45, 60). Additionally, the ratio of visceral adipose tissue/subcutaneous adipose tissue (VAT/SAT) was a predictor of mortality (42, 49), such as visceral adipose tissue/muscle area (VAT/MA) (60) and the ratio of visceral adipose tissue/total adipose tissue (VAT/TAT) being predictors of disease severity (50). Furthermore, some studies found significant associations between MM and negative outcomes. Regarding SMD, MV (41), disease severity (44), and death (41, 54) were frequent among the patients with lower values. On the other hand, higher quantities of MM were indirectly related to frequencies of mortality (60, 62), and ICU admission (7, 56, 58). Nevertheless, some authors did not find significant associations between the parameters derived from CT and worse outcomes (61, 62). Similarly, Antonarelli et al. did not find associations between pectoralis muscle quantity and density with mortality nor disease severity, and Moctezuma-Velázquez et al. did not find significant associations between skeletal muscle index (SMI) and ICU admission, MV, or mortality (48).

## 4. Discussion

The objectives of this scoping review were to determine how BC was evaluated in the studies assessing hospitalized COVID-19 patients. Our findings suggest that CT followed by BIA and US were the main assessment tools utilized in COVID-19 adult populations. Several reasons may explain this preference. Regarding the studies with CT, the radiologic tool was routinely applied in all COVID-19 patients included in the studies to check pulmonary states. Chest scans often contain the 12th thoracic vertebrae, widely reported in the included studies as the reference scan to assess BC (7, 36, 37, 48, 53, 56, 57, 59, 60, 65, 66) and the third lumbar vertebrae as well (15, 16, 35, 40, 43, 47, 49, 51, 54, 56, 63, 64, 66). The remaining studies also utilized scans but from alternative levels (34, 38, 39, 41, 42, 45, 46, 52, 55, 58, 61, 62).

### 4.1. Regarding the tools, how did the studies with COVID-19 patients evaluate body composition?

Regarding the studies included in our review, there was a discrepancy in how the protocols were reported. Although a great deal of the studies described in detail how the CT scan was performed and how they proceeded with the analysis of the images, a few articles did not report the protocols for CT scanning (16, 54, 59, 64) or the image analysis (35, 52, 65, 66), neither in the manuscript nor the supplementary material. Another finding was the incomplete exploration of the results. Some studies did not report more than one parameter of BC derived from the assessment tool in their studies (7, 46, 65). An outstanding finding of the studies included was the utilization of artificial intelligence tools to determine body compartments through CT (45). This strategy can bring a faster and more accurate data report, facilitating the work of clinicians and researchers.

Not surprisingly, BIA was not reported to be used as much as CT in the hospital setting, but its characteristics (portability, non-invasiveness, convenience, and inexpensiveness) facilitate its use in routine care and research. Some requirements are needed for the evaluation, and CT outstands as a BC assessment tool for not needing them. For BIA, there are prerequisites on body size, temperature, and fluid and electrolyte balance that must be observed before the evaluation. Failing to fulfill such requirements may compromise the results (67). COVID-19 patients, especially in the intensive care unit (ICU), do not fit most of these demands, hence impairing the assessment with BIA. It is crucial to emphasize that most studies evaluating the critically-ill did not assess FM nor FFM, but the parameters which are feasible for ICU patients, like PhA and other crude values of BIA (25, 26). Comparably to BIA, which can be used at the bedside, US stood out as the third most used BC tool.

Recently, the interest in evaluating BC through US has been increasing due to its good suitability in critically ill patients (68, 69). Since some of the hospitalized patients with COVID-19 are prone to critical illness and require MV, US can be a useful method to assess MM changes due to prolonged hospitalization, allowing clinicians to make early nutritional interventions. Thus, not surprisingly, US was used in studies with critically ill patients (6, 28, 30) as well as in studies that aimed to evaluate changes in MM during hospitalization (6, 28, 29).

### 4.2. What were the objectives of the studies with COVID-19 patients submitted to body composition assessment?

Most studies aimed to investigate the associations between the prognosis of COVID-19 and the parameters derived from the tools. However, the studies using BIA by Moonen et al. (9), Reyes-Torres et al. (26), and Ryrsø et al. (27), the studies using US by Andrade-Júnior et al. (28) and Bologna and Pone (29), and the studies using CT by Hoyois et al. (46), Faiella et al. (63), McGovern et al. (64), and Molwitz et al. (66) all had other objectives but reported data on at least one BC parameter.

It is a fact that COVID-19 manifests itself more severely, with easier infection, and with higher morbidity and mortality in those who suffer from obesity (3–5, 70, 71). This is because obesity affects most physiological processes and presents an exacerbated inflammatory state (72), worsening the immune response. Also, the degrees of obesity according to body mass index (BMI) were directly proportional to the risk for hospitalization, ICU admission, invasive MV, and in-hospital mortality (73). However, BMI alone is not the best indicator of obesity, as it does not reflect adipose tissue content nor its distribution (74), and most previous studies did not evaluate adiposity itself but an estimation that may not have provided reliable clinical data.

Besides obesity, reduced MM or low SMD were associated with a worse prognosis in patients with COVID-19 (6, 7). Some studies have shown associations between muscle quality and quantity parameters and worse results (6, 28, 30–32), using US as an evaluation tool. This shows us that it is essential to know the muscle quantity and quality of individuals. Others using the BIA tool verified the relationship of PhA (18, 20–22, 25) and percentage of FM (24, 33) with a worse prognosis. Furthermore, in more current studies, authors showed controversial results in which the amount of MM is not associated with negative results, such as frequency of mortality (56, 60) and ICU admission (56, 58).

These controversial results might have occurred due to the limitations of the evaluation tool used, since the studies that showed no relationship between the amount of MM and negative results used CT, which can be influenced by the size of the patient and tissues such as subcutaneous adipose tissue; for example, even muscles may not appear in the cross-sectional image (75).

### 4.3. What were the main findings regarding body composition parameters and COVID-19 patients?

Our results show that a great number of studies aimed to analyze the associations between BC and COVID-19 prognosis. The study by Moonen et al. was the most comprehensive, including data not only on hydration status but on VAT, MM, and FFM among others (21, 22).

BIA can estimate BC based on prediction equations, but unfortunately, the equations are used for specific populations, increasing the possibility of misestimation (76). Although 10 studies evaluated BIA and outcomes in COVID-19, most of these studies did not find an association between the BC estimation and risk of severe disease (17, 18, 21, 22). Nevertheless, Hegde et al. found the percentage of FM to be an indicator of LOS and disease severity upon admission (24). Many reasons may have contributed to the lack of evidence, e.g., small samples, utilization of inadequate equations, and non-attendance to the prerequisites of BIA evaluation among others.

In the study by Moonen et al. (22) that aimed to assess the differences in BC between ward patients (*n* = 30), and ICU patients (*n* = 24), several parameters (soft lean mass, percentage of FM, FFM, FM, dry weight, VAT area, and SMI) were assessed to find possible associations between BC and prognoses, but no significant results were found. Reliable results may be affected by the hydration state of the ICU patients.

Another study regarding BC assessment in COVID-19 patients through US (28) found that patients in a severe state had a reduction in both the cross-sectional rectus femoris muscle area and in the thickness of the anterior compartment of the quadriceps (28). MM was also a predictor of LOS in patients with moderate to severe disease in the study by Gil et al. (62), and changes in muscle parameters (echogenicity) were a predictor of mortality in the critically ill (30). In inflammatory diseases like COVID-19, impaired protein synthesis and catabolism leading to sarcopenia are associated with high CRP concentration; however, this relationship is not yet clear (11).

However, there is no data to support the validity of US to assess BC in specific populations for predicting COVID-19 prognosis (77). This could be due to the lack of standardization of the measurements and the absence of cutoff values for US parameters, e.g., the thickness of the vastus intermedius muscles and the rectus femoris, and the thickness of the quadriceps muscle layer, to evaluate the loss of MM and quality (78). Nevertheless, CT cutoff values for visceral obesity, low muscularity, muscle attenuation, and SMI were determined from many populations in the included studies. The associations between a worse prognosis and CT parameters were reported not only in original articles but also in secondary analyses.

In a meta-analysis with four studies evaluating BC and outcomes in COVID-19 patients, a higher VAT area was significantly associated with ICU admission and MV (79). Furthermore, in another meta-analysis with 539 patients utilizing CT cross-sectional images (slices), increased TAT and higher VAT areas had a significant association with COVID-19 disease severity (80).

### 4.4. What body composition alterations occurred in patients with COVID-19 during hospitalization?

A few studies evaluated the status of MM during hospitalization, and the most used tool for this assessment was the US. It was evident in three studies that the thickness of MM decreased (6, 28, 29). The loss of the tissue can be explained by a few reasons. COVID-19 patients have a combination of symptoms that may reduce nutritional intake as well as a systemic inflammation state that accelerates the MM loss during hospitalization (11, 81). Additionally, the immobilization and poor nutrition throughout the hospital stay also impair the maintenance of MM (82). Therefore, COVID-19 patients may suffer from decreased functional capacity and low physical function, as well as a hindered conduct of daily-life activities after hospital discharge (83). In the retrospective study by Bologna and Pone (29) which used US to verify the preservation of MM during hospitalization after arginine supplementation, the treated group had a significant maintenance of the MM when compared to the control group. It is important that, in clinical practice, not only must the identification of the patient’s risk for nutritional deterioration be addressed, but also the implementation of an adequate nutritional strategy. Hence, individualized, multi-modal nutritional care must be implemented from the beginning of admission (82).

This scoping review has several limitations. The first is the non-inclusion of potential scientific productions. Our searches were conducted in three different scientific literature databases and resulted in 1,220 citations and another six citations were added from the bibliography lists available in the selected articles (18, 43, 53, 54, 57, 61). These three databases cover most of the medical literature regarding BC and COVID-19. However, studies published in journals not indexed in these databases were probably not included. Furthermore, a great number of the included journal papers that evaluated the associations between BC and prognosis in COVID-19 patients had very low levels of scientific evidence due to, e.g., their small sample sizes and observational designs. Additionally, the variability between studies was high in terms of sample size, statistical analysis, methodologies applied at the moment of the evaluation, clinical conditions of the patients, and the parameters retrieved from the assessments. Notably, this could be due to the number of centers enrolled in the studies, which is also a determinant of external validity as well as the sample size estimation for each study.

Although our study presents several limitations, its strengths must be addressed. This was the first scoping review evaluating BC assessment in COVID-19 patients. Our main findings suggest that BC tools were used specially to provide predictive value to COVID-19 prognosis. Henceforth, the interrelations between BC and COVID-19 must be further investigated through original articles and secondary studies, preferably for each kind of assessment tool. Our perspectives are addressed to clinicians and researchers that may have a better overview regarding the state of the art of BC and COVID-19. Thus, health practitioners and researchers may conduct BC assessments in clinical practice or elucidate through systematic reviews better thresholds for BC in COVID-19 patients for the early detection of severity risk.

## 5. Conclusion

Our findings suggest that CT was the most common BC assessment tool, followed by BIA and US. This finding may be due to the opportunistic nature of CT, as patients had the scans to assess lung impairment during the disease. Most studies evaluated BC to find associations with adverse events, such as LOS and mortality. There is little evidence about BC changes during hospitalization. As the COVID-19 pandemic continues worldwide, new studies to be published may fill this gap in the literature.

## Author contributions

IV: methodology, data curation, writing-original draft preparation, and writing-reviewing, and editing. IS and AB: writing-reviewing and editing. AF: conceptualization, methodology, data curation, writing-original draft preparation, and writing-reviewing, and editing. All authors contributed to the article and approved the submitted version.

## Funding

This study was financed in part by the Coordenação de Aperfeiçoamento de Pessoal de Nível Superior—Brazil (CAPES)—Finance Code 001. AF received a productivity scholarship from the Brazilian National Council for Scientific and Technological Development (CNPq).

## Conflict of interest

The authors declare that the research was conducted in the absence of any commercial or financial relationships that could be construed as a potential conflict of interest.

## Publisher’s note

All claims expressed in this article are solely those of the authors and do not necessarily represent those of their affiliated organizations, or those of the publisher, the editors and the reviewers. Any product that may be evaluated in this article, or claim that may be made by its manufacturer, is not guaranteed or endorsed by the publisher.
